# Durable Acidic Oxygen Evolution Via Self-Construction of Iridium Oxide/Iridium-Tantalum Oxide Bi-Layer Nanostructure with Dynamic Replenishment of Active Sites

**DOI:** 10.1007/s40820-025-01680-w

**Published:** 2025-02-25

**Authors:** Qi Guo, Rui Li, Yanan Zhang, Qiqin Zhang, Yi He, Zhibin Li, Weihong Liu, Xiongjun Liu, Zhaoping Lu

**Affiliations:** 1 Institute of Clean Energy, Yangtze River Delta Research Institute, Northwestern Polytechnical University, Xi’an, 710072 People’s Republic of China; 2 Beijing Advanced Innovation Center for Materials Genome Engineering, State Key Laboratory for Advanced Metals and Materials, University of Science and Technology Beijing, Beijing, 100083 People’s Republic of China; 3 School of Materials Science and Engineering, Harbin Institute of Technology Shenzhen, Shenzhen, 518055 People’s Republic of China

**Keywords:** Self-construction, Nanoporous, Electronic interaction, Replenishment, Proton exchange membrane (PEM) water electrolysis

## Abstract

Proton exchange membrane (PEM) water electrolysis presents considerable advantages in green hydrogen production. Nevertheless, oxygen evolution reaction (OER) catalysts in PEM water electrolysis currently encounter several pressing challenges, including high noble metal loading, low mass activity, and inadequate durability, which impede their practical application and commercialization. Here we report a self-constructed layered catalyst for acidic OER by directly using an Ir–Ta-based metallic glass as the matrix, featuring a nanoporous IrO_2_ surface formed in situ on the amorphous IrTaO_*x*_ nanostructure during OER. This distinctive architecture significantly enhances the accessibility and utilization of Ir, achieving a high mass activity of 1.06 A mg_Ir_^−1^ at a 300 mV overpotential, 13.6 and 31.2 times greater than commercial Ir/C and IrO_2_, respectively. The catalyst also exhibits superb stability under industrial-relevant current densities in acid, indicating its potential for practical uses. Our analyses reveal that the coordinated nature of the surface-active Ir species is effectively modulated through electronic interaction between Ir and Ta, preventing them from rapidly evolving into high valence states and suppressing the lattice oxygen participation. Furthermore, the underlying IrTaO_*x*_ dynamically replenishes the depletion of surface-active sites through inward crystallization and selective dissolution, thereby ensuring the catalyst’s long-term durability.

**Supplementary Information:**

The online version contains supplementary material available at 10.1007/s40820-025-01680-w.

## Highlights

- A self-constructed catalyst with in situ formed IrO_2_/IrTaO_*x*_ bi-layer nanostructure was fabricated for high-efficiency acidic oxygen evolution reaction by using an Ir–Ta-based metallic glass as the matrix.
- The coordinated nature of the catalyst’s active site is manipulated through electronic interaction between Ir and Ta, resulting in superior activity and stability.
- The underlying amorphous IrTaO_*x*_ layer dynamically replenishes the depletion of surface Ir sites through inward crystallization and selective dissolution to ensure long-term durability.

**Supplementary Information:**

The online version contains supplementary material available at 10.1007/s40820-025-01680-w.

## Graphical abstract

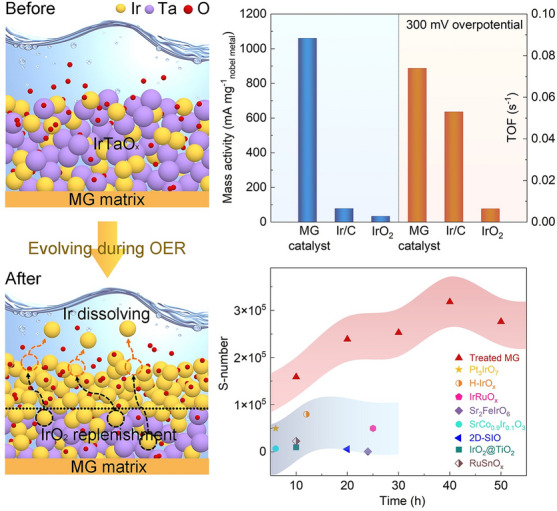

## Introduction

Green hydrogen produced through water electrolysis is considered a promising energy carrier for balancing the intermittency of renewable energy sources [1, 2]. Among various water electrolysis technologies, proton-exchange membrane (PEM) water electrolysis has recently garnered significant research interest due to its advantages of high current density, low resistance, and super gas purity [3–7], outperforming the dominant alkaline water electrolysis. However, the large-scale implementation of PEM water electrolysis is impeded by the scarcity of active and stable anodic oxygen evolution reaction (OER) electrocatalysts [8, 9]. Most existing OER catalysts are impaired by sluggish reaction kinetics in acidic environments and suffer from severe degradation under harsh corrosive and oxidative conditions [10–13]. To date, precious iridium (Ir)-based materials, such as IrO_2_, remain the only known practical OER catalysts [14–17]. Nevertheless, they still face challenges of low mass activity and thermodynamic instability during prolonged and high-current-density acidic oxygen evolution [18, 19]. Therefore, to enhance the competitiveness of PEM water electrolysis, it is highly desirable, yet remains a challenge, to develop novel acidic OER electrocatalysts with higher activity, long-term stability, and lower Ir content.

Recently, alloying strategies have emerged as a promising approach to enhancing acidic OER performance and reducing the content of Ir [20–22]. Various studies indicate that alloying or doping high-valence metals with strong electronegativity and high chemical stability, such as Ta, W, and Nb, can effectively enhance the OER activity and stability of Ir-based catalysts. For instance, synthesized compounds such as Ir_0.1_Ta_0.9_O_2.45_ [23], Ta_*x*_Tm_*y*_Ir_1−*x*−*y*_O_2−*δ*_ [24], Ir-W@Ir-WO_3−*x*_ [25], and Ir/Nb_2_O_5−*x*_ [26] demonstrate superior OER activity and stability compared to basic IrO_2_ nanoparticles in acid media due to the electronic structure manipulation induced by high-valence metals, which can effectively optimize the Ir–O bonding strength and thereby enhancing the OER electrocatalysis. However, the stability of these nanocatalysts is still limited under a low current density (e.g., 10 mA cm^−2^) due to their inevitable agglomeration, dissolution, or detachment from the support (usually the carbon) at higher current densities, which can lead to a significant decline in OER performance [27–29]. Indeed, the dissolution of active Ir species is unavoidable during acidic OER, and this issue becomes more severe under industrial current densities [30–32]. Therefore, it is highly desirable to identify an appropriate balance that can mitigate unwanted decomposition while facilitating rapid electron transfer.

To this end, herein we report a self-constructed IrO_2_/IrTaO_*X*_ bi-layer nanostructure as an efficient catalyst for acidic OER by utilizing an Ir–Ta–Ni–Nb metallic glass (MG) as the matrix. The IrTaO_*x*_ nanostructure with in situ formed catalytically active IrO_2_ nanoporous surface exhibits an ultralow overpotential of 211 mV (218 mV, experimental corrected) at 10 mA cm^−2^ and high mass activity of 1.06 A mg_Ir_^−1^ at 300 mV overpotential. Additionally, the catalyst demonstrates enhanced stability for over 650 h with negligible activity decay under a current density of 100 mA cm^−2^. The exceptional acidic OER performance outperforms that of commercial Ir/C and IrO_2_, as well as many state-of-the-art precious nanocatalysts. Experimental analyses and density functional theory (DFT) calculations reveal that the strong electronic interactions between Ir and Ta effectively modulate the coordination nature of surface lr active sites, which will prevent them from rapidly evolving to a higher valence state and dissolving. Furthermore, the underlying amorphous IrTaO_*x*_ is dynamically evolving into IrO_2_ nanocrystals to continuously replenish the surface catalytically active sites in the event of their dissolution during prolonged acidic OER operations, thereby promoting the durability and maintaining the activity of the catalyst. This work proposes an innovative strategy for designing highly efficient and stable OER alloy catalysts for practical applications in PEM water electrolysis.

## Experimental Section

### Materials

The designed Ir–Ta-based alloy ingots with different nominal compositions were prepared by melting a mixture of high-purity (99.99 wt%) elements under a Ti-gettered argon atmosphere and remelted at least five times to ensure chemical homogeneity. Then, a melt-spinning technique was used to rapidly quench the remelted alloy ingots onto the cold surface of a spinning copper wheel to fabricate the MG ribbons with a thickness of ~ 30 μm and a width of ~ 1 mm. Acidic treatment of the MG samples was carried out in an HF solution (0.5 M) for pre-activation and was then repeatedly ultrasonicated in deionized water and dehydrated alcohol to remove residual chemical substances.

### Structural Characterizations

The phase structure of the samples was determined by X-ray diffraction (XRD, Bruker D8 Advance, Cu-Kα) and grazing-incidence XRD (GIXRD, Rigaku DMAX-2500, Cu-Kα) with an incident angle of 0.5°. Microstructural characterizations were conducted using scanning electron microscopy (SEM, Zeiss Gemini 460) equipped with an energy-dispersive X-ray spectrometer (EDS), transmission electron microscope (TEM, Themis Talos F200S), and scanning TEM (STEM, Themis Z) equipped with a spherical aberration corrector. The TEM specimens were prepared using a focused-ion beam (FIB, Helios G5 UX) method. X-ray photoelectron spectroscopy (XPS, Thermo Scientific Nexsa) with an Al Kα (mono, 1486.68 eV) anode at an energy level of 72 W in a vacuum of 5 × 10^–7^ Pa was employed to probe the surface chemical state and the binding energies of the constituent elements. All binding energies were calibrated at the C 1*s* position (284.8 eV) from the contaminant carbon in the vacuum chamber of the instrument. The contents of various elements including Ir, Ta, Ni, and Nb were determined by an inductively coupled plasma-optical emission spectrometer (ICP-OES, PerkinElmer Avio 200).

### Electrochemical Measurements

All electrochemical measurements were taken on an electrochemical workstation (ParSTAT MC) using a typical three-electrode system equipped with a carbon rod as the counter electrode and a standard Ag/AgCl (3.0 M KCl) electrode as the reference electrode at room temperature. The as-spun and acid-treated MG ribbons with a fixed size of 1 mm × 5 mm × 30 μm were directly used as the working electrodes. The electrocatalytic measurements were conducted in an acidic electrolyte of 0.5 M H_2_SO_4_ aqueous solution. The commercial IrO_2_ and Ir/C (20 wt%) catalyst inks were prepared and loaded on a glass carbon electrode (diameter: 4 mm), respectively. The mass loading of these precious catalysts was about 0.2 mg cm^−2^. For convenience, all potentials in this study were converted to reversible hydrogen electrode (RHE) by the Nernst equation of *E*_RHE_ = *E*_Ag/AgCl_ + 0.197 + 0.0591 × pH and the overpotential (*η*) for OER was calculated using the equation of: *η* = *E*_RHE_ − 1.23. Electrochemical impedance spectroscopy (EIS) spectra were obtained in a frequency range of 10^5^ Hz to 0.1 Hz with an amplitude of 10 mV. All linear scan voltammetry curves were conducted at a scan rate of 5 mV s^−1^. Before each linear sweep voltammetry (LSV) test, the catalyst electrodes were measured with several cyclic voltammetry (CV) cycles until a stable CV curve was obtained. The *i*R correction of polarization curves was performed using the solution resistance estimated from EIS measurements. The potential was then corrected using the equation of *E*_iR-corrected_ = *E* – *iR*, where *i* is the current and *R* is the uncompensated electrolyte Ohmic resistance measured by EIS. The mass activity of the catalysts was calculated using the equation of *j*_mass_ = *i*_geo_/(*m*_act_ × Ir_wt%_), where *i*_geo_ is the geometric current obtained from LSV, *m*_cat_ is the mass of the catalytic layer, and Ir_wt%_ is the mass ratio of Ir. The turnover frequency (TOF) was calculated using the equation of $\text{TOF}=\frac{\left|j\right|\times {A}_{\text{geo}}\times {N}_{A}/(4F)}{\text{ECSA}/{S}_{a}}$, where *j* is the geometric current density, *A*_geo_ is the geometric area of the catalytic surface, *N*_A_ is the Avogadro constant (6.022 × 10^23^ mol^−1^), *F* is the Faraday constant (96,485.3 C mol^−1^), ECSA is the electrochemically active surface area of the catalyst, and *S*_a_ is the average area of each active site [33]. The ECSA was calculated using the equation of ECSA = *C*_dl_/*C*_S_, where *C*_dl_ is the electrical double-layer capacitor and is measured from double-layer charging curves using cyclic voltammograms in a non-Faradaic region with scan rates ranging from 10 to 100 mV s^−1^. *C*_S_ is the specific capacitance of the sample (set as 40 μF cm^−2^ according to the previous reports) [34]. The chronopotentiometry tests under the different current densities ranging from 100 mA cm^−2^ to 1 A cm^−2^ were carried out to evaluate the long-term durability of the catalyst. ICP measurement was used to determine the dissolved elements in the electrolyte after the durability test. The S-number was calculated using the equation:$S\text{-number}=\frac{{n}_{{o}_{2}}}{{n}_{\text{Ir}}}$, where $n_{Ir}$ is the total amount of Ir dissolved in the electrolyte, ${n}_{{o}_{2}}$ is the amount of oxygen molecules produced during a 650-h test at 100 mA cm^−2^. This value was calculated according to Faraday's laws of electrolysis of ${n}_{{o}_{2}}=\frac{j\times s\times t}{z\times F}$, where *j* is the geometric current density, *s* is the geometry area of the electrode, *t* is the electrolysis time, *z* is the number of electrons transferred to produce an oxygen molecule, and *F* is the Faraday constant.

Isotope-labeled differential electrochemical mass spectrometry (DEMS) measurements were taken in an electrochemical cell with a typical three-electrode system equipped with an operando DEMS system (QAS-100, Linglu Instruments), where the MG catalyst samples with ^18^O-labeling were used as the working electrodes, an Ag/AgCl electrode prefilled with saturated KCl aqueous solution was used as the reference electrode, and a Pt plate was used as the counter electrode. CV measurement was taken in ^16^O 0.5 M H_2_SO_4_ electrolyte with a scan rate of 5 mV s^−1^. The gaseous products including ^32^O_2_, ^34^O_2_, and ^36^O_2_ were probed in real time by the mass spectrometer.

### Theoretical Calculation

All calculations were carried out in the framework of DFT using the Vienna Ab initio Simulation Package (VASP) [35–37]. The generalized gradient approximation (GGA) with the Perdew–Burke–Ernzerhof (PBE) function was used to describe the exchange–correlation energy [38]. The projected augmented wave (PAW) method and pseudopotentials were used to describe the interactions between valence electrons and ions [39]. To ensure the efficiency of the computational results and parallel computing, a 2 × 2 × 3 k-point grid under Monkhorst–Pack with a truncation energy of 450 eV is used in the optimization process. To correct the localization effect of D-orbital electrons in transition metal atoms, the value of efficient Hubbard U was set as 3.0 eV for both Ir and Ta. The lattice parameters and ionic positions of all crystals were fully relaxed, and the convergence criteria for the total energy of all relaxed atoms and the final force were 10^–5^ eV and 0.03 eV Å^−1^, respectively.

## Results and Discussion

### Catalyst Fabrication and Acidic OER Performance

**Fig. 1:**
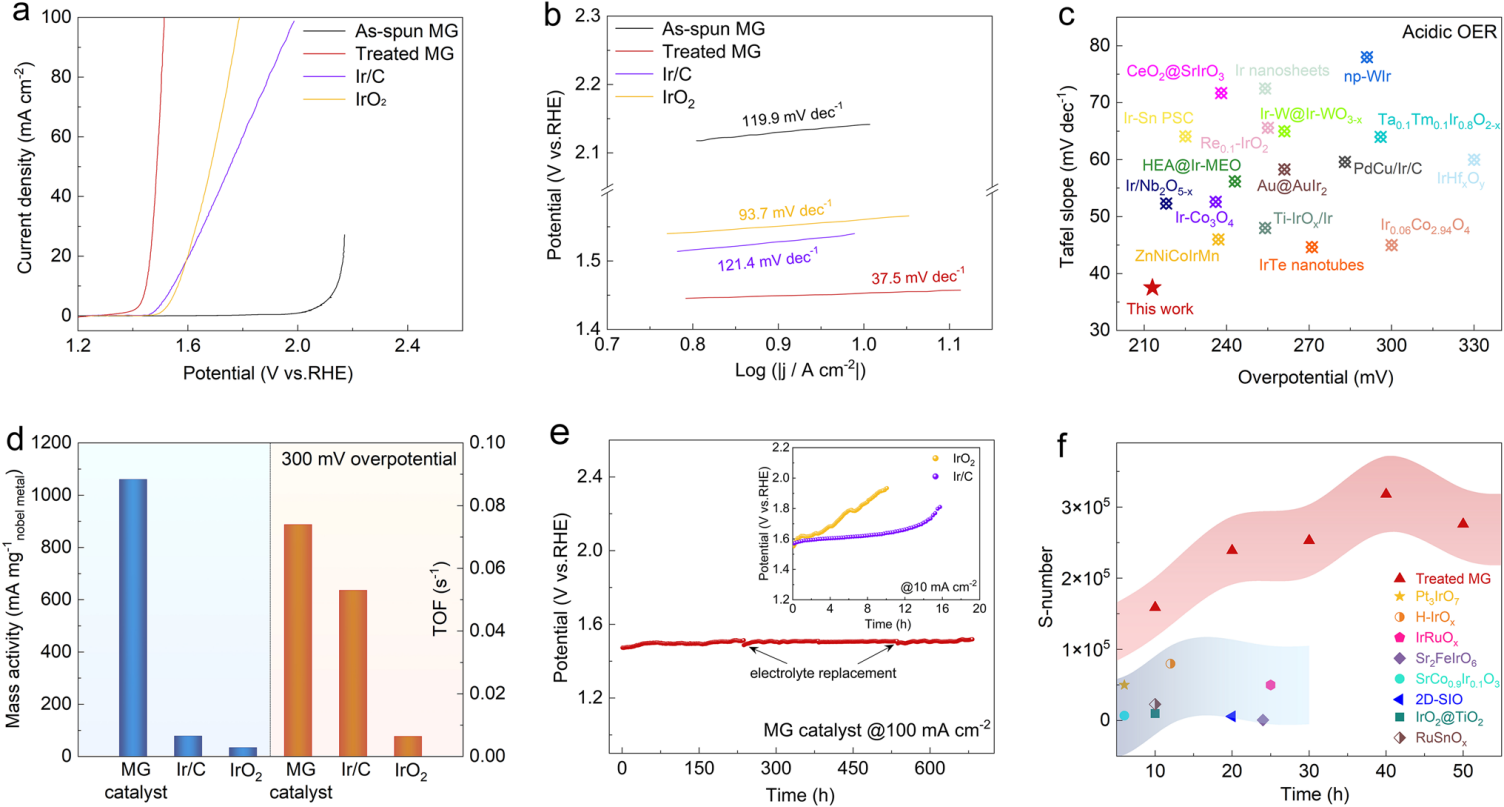
Electrochemical OER performance in acid. **a** Linear polarization curves of as-spun Ir_30_Ta_35_Ni_29_Nb_6_ MG, acid-treated Ir_30_Ta_35_Ni_29_Nb_6_ MG, commercial IrO_2_, and Ir/C in a 0.5 M H_2_SO_4_ electrolyte. **b** The corresponding Tafel slopes. **c** Comparison of overpotential at 10 mA cm^−2^ and Tafel slope with recently reported Ir-based OER electrocatalysts in acid. **d** Mass activity and TOF of the Ir_30_Ta_35_Ni_29_Nb_6_ MG catalyst and commercial Ir/C and IrO_2_ at a 300 mV overpotential. **e** Chronopotentiometry curve of the MG catalyst at a current density of 100 mA cm^−2^. The inset shows the stability test results for the commercial Ir/C and IrO_2_ catalysts at a current density of 10 mA cm^−2^. **f** Comparison of calculated S-number of the MG catalyst with various recently reported electrocatalysts during 50-h OER stability test

Ir_30_Ta_35_Ni_29_Nb_6_ MG matrix was prepared using a melt-spinning method. XRD pattern reveals the absence of any crystalline phase (Fig. S1), confirming the amorphous nature of the as-spun Ir_30_Ta_35_Ni_29_Nb_6_ MG precursor. Subsequently, an acid treatment was performed to create a catalytically active surface on the MG matrix. It is noted that the pre-activation process is crucial for enhancing the electrochemical activity without compromising the amorphous structure and mechanical flexibility of the MG matrix (Fig. S2). The electrocatalytic OER performances of the as-spun MG precursor and acid-treated MG were then evaluated in a 0.5 M H_2_SO_4_ electrolyte and compared with commercial Ir/C and IrO_2_. In Fig. 1a, the LSV curves demonstrate that the acid-treated MG achieves an overpotential of 211 mV at a current density of 10 mA cm^−2^ (the current normalized to geometric surface area), significantly lower than that of its initial counterpart (901 mV) and the commercial catalysts (312 mV for Ir/C and 330 mV for IrO_2_, respectively). To obtain a more accurate overpotential, we adjusted the value using experimental corrections [40] (Fig. S3). The correction results indicate that the overpotential of the MG catalyst is 218 mV, with only minor fluctuations compared to the theoretical overpotential of 211 mV. The corresponding lowest Tafel slope of 37.5 mV dec^−1^ in Fig. 1b further demonstrates the superior electrochemical kinetics of the acid-treated MG. In addition, these characteristic values were compared with various Ir-based OER electrocatalysts recently reported (Fig. 1c and Table S1). Encouragingly, compared to many state-of-the-art precious nanocatalysts, the MG catalyst exhibits superior acidic OER activity. We then estimated TOF of these catalysts, which represents the number of oxygen molecules evolved per second from each active site at a specific overpotential [41]. As illustrated in Fig. 1d, the TOF value of the MG catalyst at an overpotential of 300 mV is higher than that of commercial Ir/C and IrO_2_, demonstrating the superior intrinsic activity of the catalytic Ir sites. Additionally, the faster charge transfer kinetics measured by EIS (Fig. S4) and the higher specific activity normalized by ECSA (Figs. S5 and S6) further validate the intrinsically improved OER activity of the acid-treated MG. More specifically, as a self-supporting material, the catalyst surface delivers a remarkable mass activity of 1.06 A mg_Ir_^−1^ at an overpotential of 300 mV, which is 13.6 and 31.2 times relative to that of commercial Ir/C (78.7 mA mg_Ir_^−1^) and IrO_2_ (34.3 mA mg_Ir_^−1^), respectively (Fig. 1d). This value also exceeds that of most Ir-based nanoparticle catalysts (Table S2), demonstrating that the MG catalyst not only possesses enhanced OER activity but also offers a cost advantage by reducing the amount of Ir usage.

In addition to high electrocatalytic activity, the operational stability of the catalyst is also a crucial metric for PEM water electrolysis. The OER durability of the MG catalyst in acid was assessed using a chronopotentiometry test at a constant current density of 100 mA cm^−2^. As illustrated in Fig. 1e, the catalyst demonstrates negligible performance degradation for over 650 h, with an overpotential degradation rate of approximately 10 μV h^−1^, which is lower than the latest specification (14 μV h^−1^) for the PEM water electrolyzer. Notably, there is an increase in voltage of approximately 15 mV during the first 30 h of OER. This slight elevation can be attributed to the unstable electrochemical environment during the initial stages of the electrolysis system. The superior stability of the MG catalyst during the long-term stability test was further validated by the comparable LSV curves obtained after 200 and 650 h of OER (Figs. S7 and S8). In contrast, the commercial IrO_2_ and Ir/C catalysts exhibit rapid increases in overpotential (approximately 10 and 16 h at 10 mA cm^−2^, respectively) due to catalyst detachment resulting from dissolution (see inset in Fig. 1e). Furthermore, the concentration of dissolved metal ions in the electrolyte was detected by ICP-OES (Fig. S9). This measurement indicates that, although the dissolution of Ir is inevitable under acidic OER conditions, the dissolution rate of Ir (0.125 μg h^−1^) in the MG catalyst is much lower than that of commercial IrO_2_ and Ir/C. These results strongly demonstrate the excellent acidic OER stability of the catalyst. Additionally, we employed a stability parameter known as the stability number (i.e., the S-number) to evaluate the chemical stability of the catalyst, which is defined as the ratio between the number of oxygen molecules and the number of dissolved Ir cations [42]. As illustrated in Fig. 1f, the S-number of the MG catalyst was calculated to be as high as 2.7 × 10^5^ at the initial state (≤ 50 h), which is superior to many recently reported Ir-based electrocatalysts [19, 43–49], such as IrO_2_@TiO_2_ nanoparticles (1.0 × 10^4^), SrCo_0.9_Ir_0.1_O_3_ (7.0 × 10^3^), and 2D-SIO (6.0 × 10^3^). Furthermore, even after the extended durability test (i.e., 650 h), the S-number value remains high (5.4 × 10^4^ to 9.0 × 10^4^) (Fig. S10). This measurement indicates that the catalytic structure is stable during the long-term acidic OER operation. It is also noteworthy that even at an ampere-level current density of 1 A cm^−2^, the overpotential of the catalyst remains as low as approximately 450 mV for more than 100 h (Fig. S11). Furthermore, under industrial conditions of 2 A cm^−2^ at 80°C, the MG catalyst maintains high stability without significant performance degradation during OER, and its S-number was calculated to be as high as 1.16 × 10^4^ (Fig. S12). The outstanding OER performances under these industrial-relevant conditions further highlight its enormous potential for practical applications in PEM water electrolysis.

### Structural Origins for the Enhanced OER Activity and Stability

**Fig. 2:**
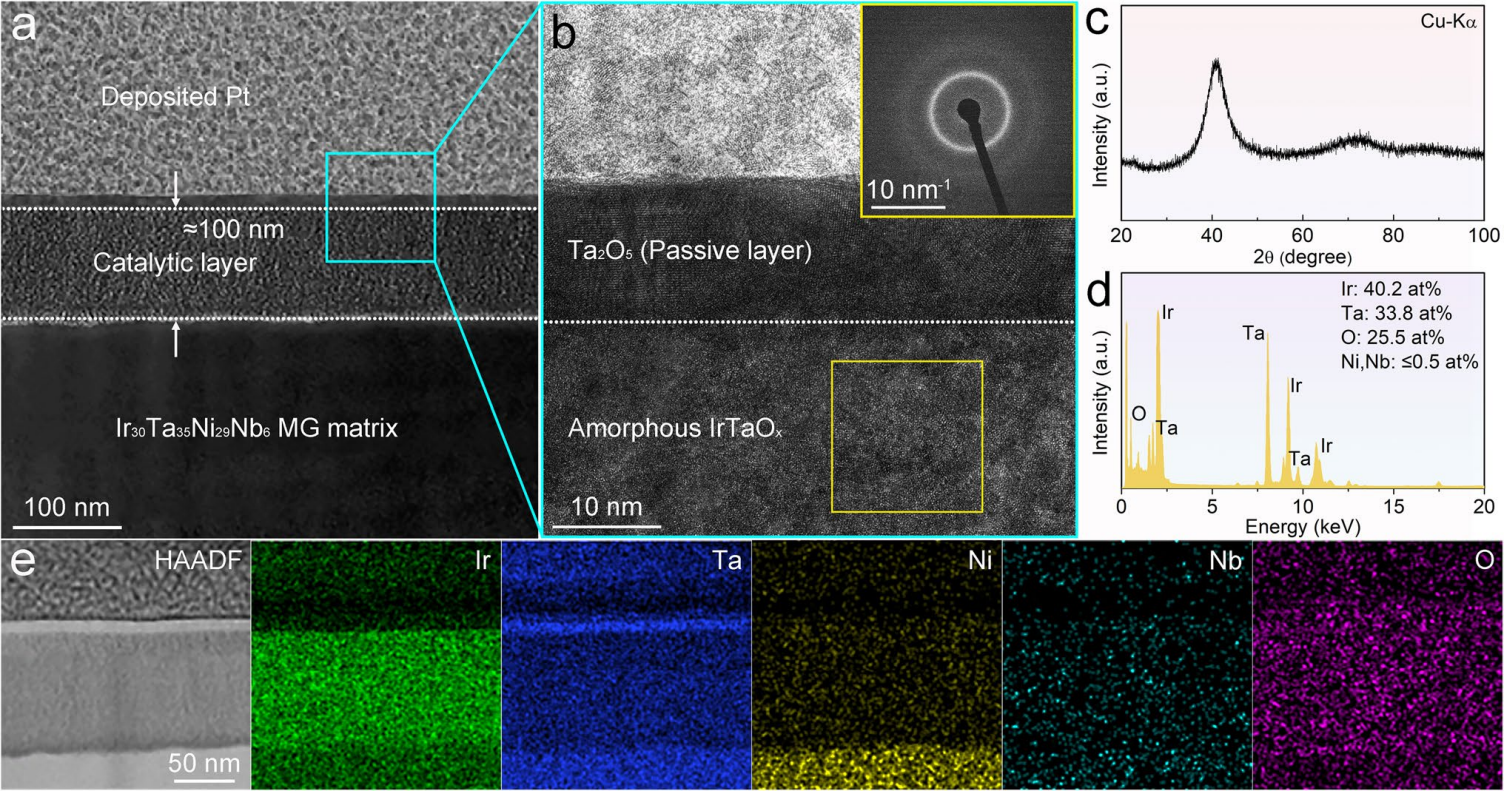
Characterization of the surface catalytic layer structure. **a** Cross-sectional TEM image of the acid-treated Ir_30_Ta_35_Ni_29_Nb_6_ MG (The top layer exhibits the FIB-deposited Pt nanoparticles). **b** HRTEM image of the layer structure marked in **a**. The inset shows the SAED pattern of the region marked by the yellow square. **c** Surface XRD pattern of the IrTaO_*x*_ amorphous structure. **d** TEM-EDS analysis of the amorphous IrTaO_*x*_ layer. **e** High-angle annular dark-field (HAADF)-STEM image of the layer structure and the corresponding EDS elemental mapping

As previously mentioned, the MG catalyst demonstrates exceptional acidic OER activity and stability. This raises an intriguing question regarding whether any components of the acid-treated MG structure contribute to enhancing the electrocatalytic activity and stability. To address this issue, comprehensive structural characterizations of the catalyst were performed. Figure 2a presents the cross-sectional TEM image of the acid-treated MG, which reveals an etched layer with a thickness of approximately 100 nm formed on the Ir_30_Ta_35_Ni_29_Nb_6_ MG matrix after acidic treatment. The high-resolution TEM (HRTEM) in Fig. 2b, along with the corresponding selected area electron diffraction (SAED) pattern (the inset in Fig. 2b), demonstrates an amorphous nature of the etched layer, consistent with the grazing incident XRD characterization (Fig. 2c). The TEM-EDS profile shows that the surface Ni and Nb elements were selectively etched away, resulting in the formation of an IrTaO_*x*_-layered nanostructure (Fig. 2d). Notably, a small number of Ir–Ta-based nanocrystals was also observed within the amorphous structure (Fig. S13). This phenomenon occurs because surface corrosion under strongly acidic oxidation conditions may lead to the partial transformation of the metastable amorphous structure into a crystalline state. Moreover, it is seen that a compact passivated Ta_2_O_5_ crystalline layer with a thickness of approximately 15 nm formed on the surface of the amorphous IrTaO_*x*_ layer. From the perspective of reaction kinetics, the corrosion mechanism of the as-spun MG in HF can be described as a diffusion process between metal elements and the medium in the HF solution (i.e., F^−^, O^2−^, and OH^−^) [50]. The catalytically active IrTaO_*x*_ nanostructure forms on the MG matrix due to the preferential etching of Ni and Nb by F^−^ and the oxidation of Ir and Ta by O^2−^, resulting in improved OER activity and a 4-day-acid-treated MG yields the best performance (Figs. S14 and S15). As the treatment time is further extended, elemental Ta readily reacts with O^2−^ and OH^−^ to generate a passivation layer on the surface [51–53]. The gradually formed Ta_2_O_5_ passivation layer protects the IrTaO_*x*_ layer and the MG matrix from excessive corrosion, thereby stabilizing the layered catalyst structure. The STEM elemental mapping analyses (Fig. 2e) further confirm the formation of the composite oxide layers on the MG matrix and the elemental distributions.

**Fig. 3:**
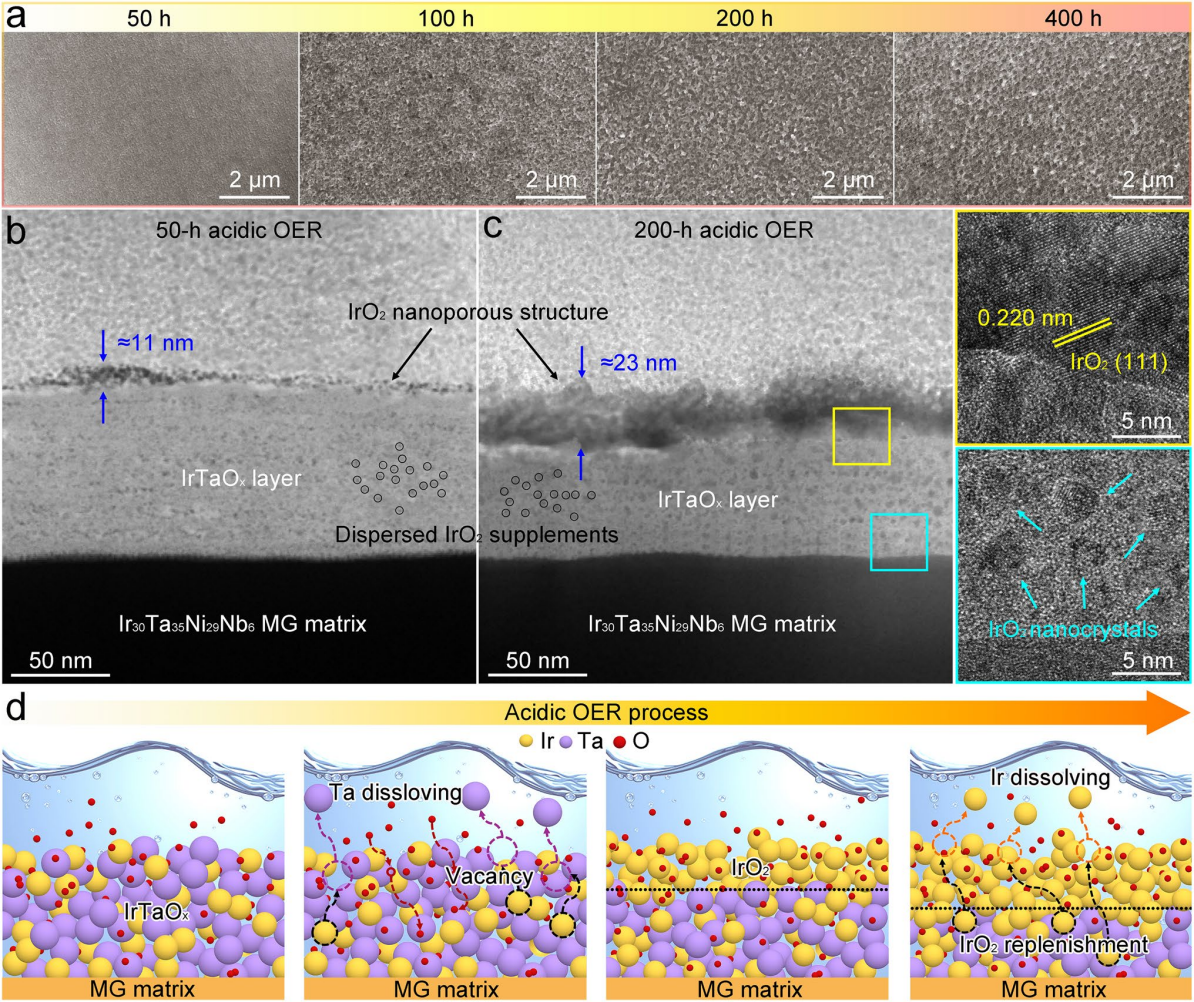
Dynamical structure evolution during acidic OER. **a** Ex situ surface SEM images for the formation of a nanoporous IrO_2_ surface structure under acidic OER conditions. **b** TEM image of the IrTaO_*x*_ layer structure after 50-h acidic OER. **c** TEM image of the IrTaO_*x*_ layer structure after 200-h acidic OER. The insets show the corresponding HRTEM images of the selected areas marked in **c**. (yellow square: top right; blue square: bottom right). **d** Schematic diagram illustrating the evolution of the IrTaO_*x*_ layer structure and the replenishment of surface IrO_2_ during OER

We assume that the amorphous IrTaO_*x*_ nanostructure is the reason for the enhanced OER performance of the MG catalyst. To verify this hypothesis, we investigated the structural evolution of the catalyst during long-term acidic OER. As illustrated by the change in surface morphology with reaction time (Fig. 3a), a nanoporous structure gradually developed on the IrTaO_*x*_ surface, stabilizing into a bi-continuous nanoporosity with an average size of approximately 100 nm. Even after 400 h, the nanoporous structure remained almost unchanged, indicating its extraordinary stability during the acidic OER catalysis. It should be noted that under the highly acidic and strongly oxidizing conditions of OER, the passivating Ta_2_O_5_ layer on the surface of IrTaO_*x*_ nanostructures is expected to dissolve rapidly and disappear during the initial stages due to its electrochemical corrosion behavior under acidic OER conditions (Fig. S16). Figure 3b, c, respectively, presents the changes in the IrTaO_*x*_ layer after 50- and 200-h OER catalysis characterized by TEM. Notably, a unique bi-layer structure emerged during the OER process, characterized by the formation of a thin upper layer with a thickness of approximately 13 nm on top of the IrTaO_*x*_ layer. The thickness of this layer remained constant at about 23 nm, exhibiting an obvious nanoporosity after 200 h, consistent with the SEM observation. The HRTEM image of the nanoporous layer (the top right inset in Fig. 3c) reveals the lattice spacing for the (111) plane of IrO_2_, confirming the formation of IrO_2_ nanocrystals. This finding is further supported by the STEM elemental mapping analysis of the reacted layer structure (Fig. S17). These characterizations demonstrate that catalytically active nanoporous IrO_2_, with increased accessible contact areas, can be directly self-constructed on IrTaO_*x*_ through acidic oxygen evolution, providing abundant active sites for electrocatalytic reactions. Additionally, considerable numbers of nascent IrO_2_ nanocrystals were observed to be dispersed within the IrTaO_*x*_ layer below (see the right bottom inset in Figs. 3c and S18), indicating further crystallization of the amorphous IrTaO_*x*_ layer during the OER process. Moreover, the thickness of the IrTaO_*x*_ layer decreased after long-term OER (i.e., reduced by approximately 24 nm after 200 h), confirming that the surface IrO_2_ nanocrystals primarily originate from the underneath IrTaO_*x*_. Given the inevitable dissolution of the surface Ir active species during OER (Fig. S9), it is reasonable to infer that the amorphous IrTaO_*x*_ layer acts as a reservoir through inward crystallization and selective dissolution, allowing the newly formed IrO_2_ nanocrystals to dynamically compensate for the loss of the surface-active IrO_2_. In this case, the underlying IrTaO_*x*_ significantly influences the number of active sites available for electrocatalysis. The increase in the thickness of the IrO_2_ nanoporous layer on the catalyst surface is accompanied by a decrease in the thickness of the IrTaO_*x*_ layer following prolonged OER. Consequently, the total number of active sites remains relatively stable due to a dynamic replenishment of the underlying IrTaO_*x*_, even as the IrO_2_ nanoporous layer continues to thicken. This mechanism provides a replenishment capability that helps maintain the balance of activity and stability on the catalyst surface.

The dynamic evolving process is further illustrated in Fig. 3d, which mainly encompasses the diffusion, oxidation, and dissolution of the constituent elements. Under acidic and oxidative conditions, the significant accumulation of oxygen atoms on the surface of the metastable amorphous IrTaO_*x*_ facilitates the inward diffusion of oxygen. Meanwhile, non-noble Ta elements dissolve outward due to their relatively lower corrosion resistance compared to Ir, resulting in numerous vacancies on the surface. Consequently, Ir atoms tend to diffuse and aggregate to fill these vacancies due to the chemical potential gradient and form IrO_2_ by binding with oxygen, thereby resulting in the observed two-layer structure. With continuous oxygen evolution, the amorphous IrTaO_*x*_ layer is expected to undergo further crystallization and dynamic evolution, particularly in response to the dissolution of IrO_2_ reacted on the surface. Based on these experimental data, it is evident that the self-constructed bi-layer structure on the MG matrix is crucial for enhancing OER performance, where the IrO_2_ nanoporous layer generated on the surface provides a high specific surface area that facilitates rapid mass transfer, which contributes to maintaining long-term OER performance. Additionally, the underlying IrTaO_*x*_ layer can dynamically evolve into IrO_2_ nanocrystals through crystallization and selective dissolution, replenishing the depletion of surface Ir active sites and balancing OER activity and stability in acid media.

As is well known, exposed IrO_2_ active species play a crucial role in the intrinsic OER property. However, the contribution of the Ta element in the catalyst remains unclear. To understand this, an Ir_30_Ni_42_Nb_28_ MG precursor without Ta alloying was prepared for comparison and subjected to the same acid treatment (Figs. S19 and S20). Notably, a nanoporous IrO_2_ surface was directly formed on the Ir_30_Ni_42_Nb_28_ MG matrix due to the similar corrosion rates of Ni and Nb (Fig. S21). Compared to the MG catalyst featuring an IrTaO_*x*_ surface structure, the acid-treated Ir_30_Ni_42_Nb_28_ MG exhibits inferior activity toward acidic OER (Fig. S22). Moreover, during long-term OER operation at 100 mA cm^−2^, a rapid increase in overpotential was observed in the Ir_30_Ni_42_Nb_28_ MG catalyst after only 80 h (Fig. S23). The dissolution of Ir in the electrolyte indicates that its dissolution rate is 30 times higher than that of the Ir_30_Ta_35_Ni_29_Nb_6_ MG catalyst (Fig. S24). Furthermore, structural changes observed in the Ir_30_Ni_42_Nb_28_ MG catalyst after the stability test suggest that the surface catalytic sites were depleted, leading to a complete collapse of the catalyst structure after 80 h due to the absence of the IrTaO_*x*_ replenishment layer (Fig. S25). The absence of the Ta alloying effect results in the over-oxidative dissolution of surface IrO_2_ in strongly acidic and oxidative environments. Additionally, the lack of a dynamic complementary effect from the underlying IrTaO_*x*_ structure leads to the formation of numerous vacancies and defects on the catalyst surface, increasing susceptibility to localized corrosion. Stress concentration in the corroded areas serves as a source for crack initiation, ultimately resulting in localized spallation fractures of the catalyst surface [54]. As IrO_2_ dissolves and detaches from the catalyst surface, the oxidation rate of the MG matrix accelerates, leading to the collapse of the overall structure and a decline in OER performance. The EDS results for the Ir_30_Ni_42_Nb_28_ MG catalyst after the stability test indicate a decrease in the atomic ratio of the Ir element, with the catalyst becoming completely oxidized (Table S4). Moreover, we also evaluated the stability of the acid-treated Ir_65_Ni_29_Nb_6_, which has a higher Ir content (Fig. S26). During the 50-h OER test, the catalyst's voltage increased, indicating a decline in catalytic performance. The deep purple color of the electrolyte after 50 h of OER further suggests that Ir is rapidly dissolving due to peroxidation in the absence of Ta. These results clearly demonstrate that the Ta element is essential for the formation of the IrO_2_/IrTaO_*x*_ bi-layer structure and plays a significant role in enhancing stability during acidic OER.

### Electronic Structure Analysis and Reaction Mechanism

**Fig. 4:**
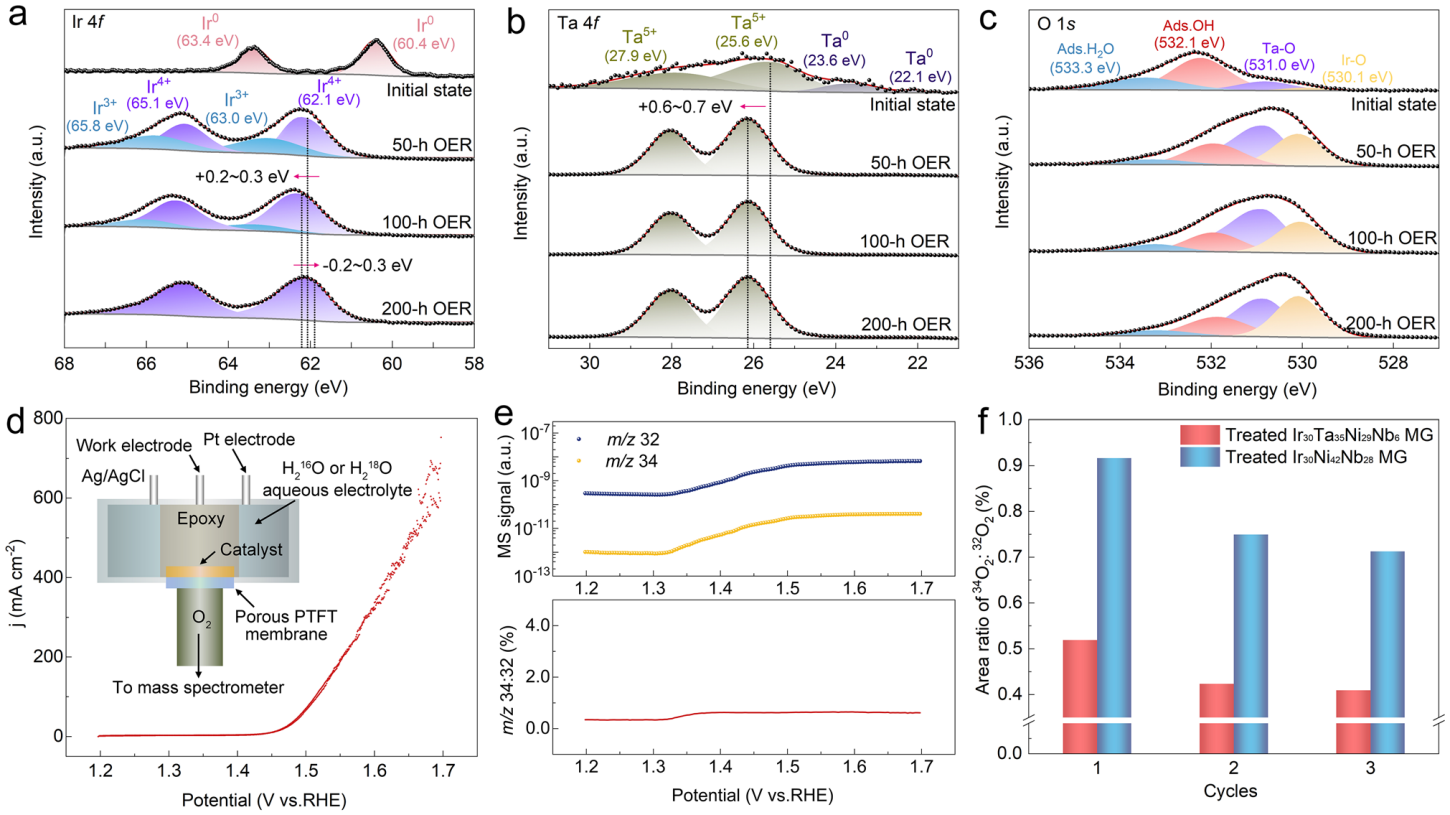
XPS analysis and isotope-labeled operando DEMS characterization. **a–c** XPS spectra of **a** Ir 4*f*, **b** Ta 4*f*, and **c** O 1*s* during the long-term acidic OER stability test (at 0, 50, 100, and 200 h). **d** CV cycle of the MG catalyst measured by DEMS. The inset shows the schematic illustration of the operando DEMS. **e** Top: DEMS signals of ^32^O_2_ (^16^O + ^16^O) and ^34^O_2_ (^16^O + ^18^O) collected during CV cycle; Bottom: The mass spectroscopy peak area ratio of ^34^O_2_/^32^O_2_ with increase in potential. **f** The ratio of ^34^O_2_/^32^O_2_ for the Ir_30_Ta_35_Ni_29_Nb_6_ and Ir_30_Ni_42_Nb_28_ MG catalysts in H_2_^16^O/0.5 M H_2_SO_4_ electrolyte during 3 OER cycles

Given the critical role of Ta alloying in affecting OER performance, it is essential to further evaluate its electronic interaction with Ir during OER [55–57]. The surface chemical state of the catalyst was examined using high-resolution XPS. Figure 4a illustrates the variation in the Ir 4*f* spectra on the MG catalyst surface during the OER process from 0 to 200 h. After 50 h, the metallic Ir was completely transformed into oxides compared to its initial state. Specifically, the oxidation states of Ir can be attributed to the Ir^IV^ and Ir^III^ species, which are located at 62.1/65.1 and 63.0/65.8 eV, respectively. It should also be noted that due to anodic polarization, the binding energy of Ir shifts positively by 0.2 to 0.3 eV after 100 h of OER, which may facilitate the further conversion of Ir^III^ to Ir^IV^. As the reaction progressed to 200 h, significant changes were observed in the peak areas of the Ir^IV^ and Ir^III^ species, with Ir^III^ completely converted into Ir^IV^. Moreover, the Ir 4*f* spectra reveal a counterintuitive decrease in the binding energy of Ir^IV^ by approximately 0.2 to 0.3 eV, indicating that the formed Ir^IV^ species are stable and would not be over-oxidized to higher oxidation states (i.e., Ir^V^ and Ir^VI^). In fact, recent studies have demonstrated that the pathways for the OER-triggered dissolution of Ir primarily involve the formation of soluble Ir^III^ or hypervalent Ir (> IV) species [58, 59]. Therefore, the surface-stabilized Ir^IV^ species in our case should account for the high chemical stability. We also characterized the variations in the Ta 4*f* spectra. As shown in Fig. 4b, the difference in XPS peaks reveals the complete transformation of the metallic Ta^0^ to oxidized Ta^V^ during the acidic OER. Additionally, the binding energy of Ta^V^ exhibits a positive shift of approximately 0.6 to 0.7 eV relative to its initial state, indicating the electronic modulation of Ir by Ta during OER. The high valence oxidation of Ta caused by the strong electronic interactions between Ir and Ta can adsorb excess oxygen atoms and prevent active Ir oxides from further oxidizing to higher valence states (e.g., the unstable Ir^V^ or Ir^VI^ species) during acidic OER, thereby enhancing the durability of the catalyst.

Figure 4c further illustrates the variation in the O 1*s* XPS spectra during the acidic OER process. The core-level spectra can be deconvoluted into three distinct oxygen species: adsorbed H_2_O, adsorbed OH, and lattice oxygen. Furthermore, to differentiate the lattice oxygen bound to various metals, the species at 531.0 eV were identified as Ta-O, while the peak near 530.1 eV is attributed to Ir–O [60, 61]. In this context, Ta atoms, which possess lower electronegativity, are expected to preferentially bind with oxygen intermediates, thereby minimizing electron loss of Ir and inhibiting the further oxidation of the surface-active Ir oxides to soluble high-valence counterparts. Additionally, it was observed that the area ratio of lattice oxygen to adsorbed OH on the catalyst surface increased after prolonged OER testing. This trend indicates that the surface-adsorbed oxygen is less susceptible to protonation and is more likely to react with H_2_O in the electrolyte to form hydroperoxide intermediates and then produce O_2_, indicating a preference for the adsorbate evolution mechanism (AEM) over the lattice oxygen mechanism (LOM) [62]. To experimentally validate this AEM pathway, isotope-labeled operando DEMS measurements were conducted (see the schematic diagram in the inset of Fig. 4d). Initially, the catalyst surface was labeled with ^18^O by conducting OER under a constant current density in an H_2_^18^O/0.5 M H_2_SO_4_ electrolyte. Then, the ^18^O-labeled MG catalyst was thoroughly washed with H_2_^16^O to eliminate any adsorbed H_2_^18^O, followed by OER cycles in an H_2_^16^O/0.5 M H_2_SO_4_ electrolyte (Fig. 4d). As the OER current increased, gaseous oxygen products including ^32^O_2_ (^16^O^16^O), ^34^O_2_ (^16^O^18^O), and ^36^O_2_ (^18^O^18^O) were detected by online mass spectroscopy (Fig. 4e). It is worth noting that the signal ratio of ^34^O_2_ to ^32^O_2_ within the OER potential range remained nearly constant at a low value of approximately 0.53%, indicating that the LOM pathway did not significantly occur on the catalyst surface. Moreover, it is important to highlight that the value of ^34^O_2_/^32^O_2_ generated on the MG catalyst is much lower than that of the reference MG without Ta alloying (i.e., the Ir_30_Ni_42_Nb_28_ MG catalyst) (Figs. 4f and S27), suggesting an inhibitory effect of lattice oxygen participation on the IrO_2_/IrTaO_*x*_ catalytic surface. The aforementioned DEMS results demonstrate that the incorporation of Ta effectively inhibits the participation of lattice oxygen during acidic OER, thereby stabilizing the surface IrO_2_ and enhancing stability.

**Fig. 5:**
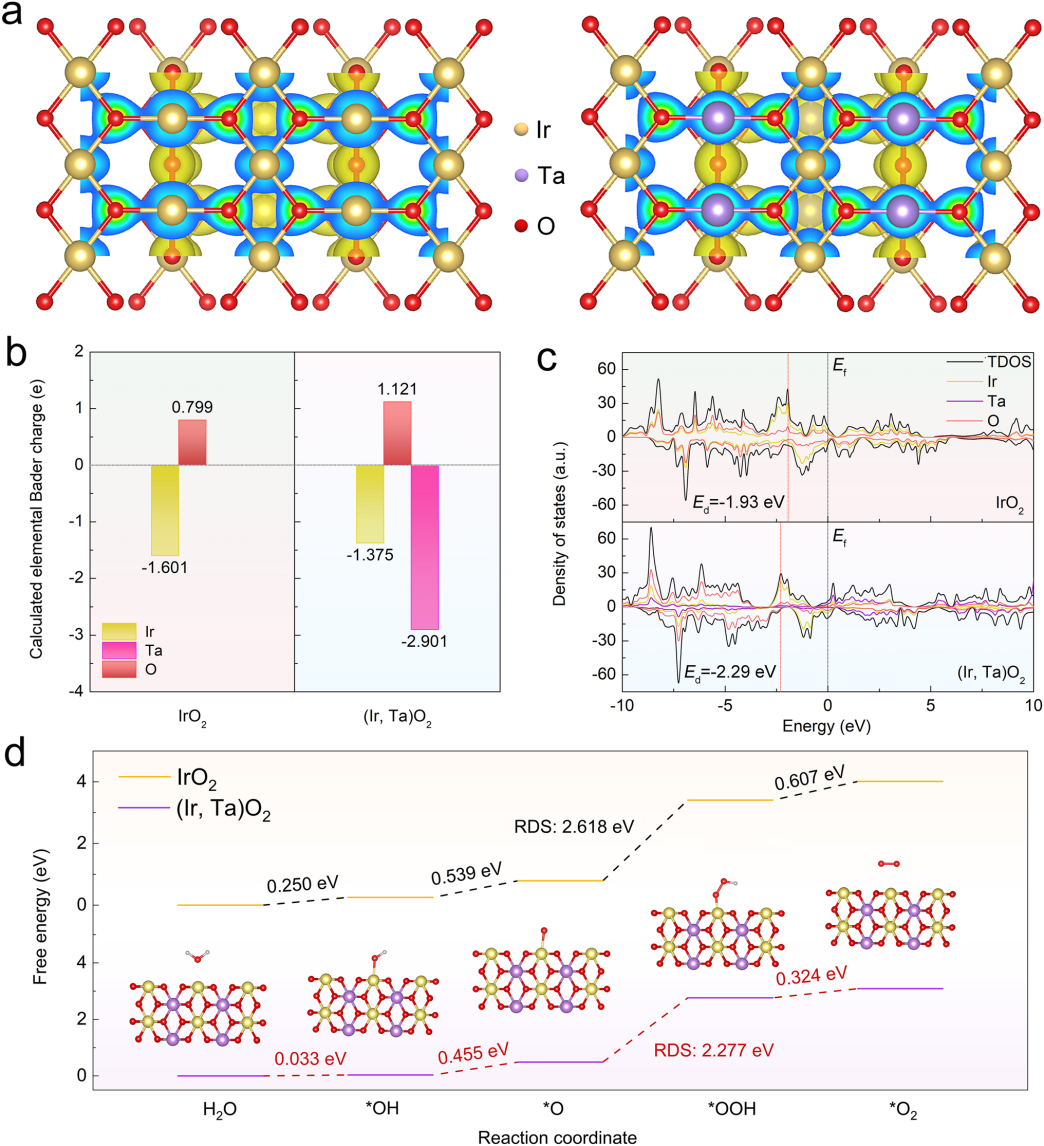
DFT calculation. **a** Charge density distribution of the (110) crystal plane for (Ir, Ta)O_2_ and IrO_2_ models. **b** Bader charges distribution of the modulated IrO_2_ and (Ir, Ta)O_2_ structure. **c** Calculated PDOS of IrO_2_ and (Ir, Ta)O_2_. *E*_*f*_ represents the Fermi energy level and *E*_d_ represents the *d*-band center. **d** OER free energy diagram for (Ir, Ta)O_2_ and IrO_2_ at a zero applied potential

We then performed DFT calculations to gain deeper insight into the impact of Ta electronic modulation on catalyst stability. For the convenience of this analysis, a simplified (Ir, Ta)O_2_ structure was modeled to simulate the catalytic surface based on the TEM-EDS result and surface ICP analysis (Table S3). Subsequently, the model was compared to the pristine IrO_2_ crystal (Fig. S28). Figure 5a illustrates the charge density distribution of the (110) crystal plane for both (Ir, Ta)O_2_ and IrO_2_, demonstrating that Ta atoms act as electron donors. The corresponding Bader charge distribution further indicates that, after Ta alloying, the donating electrons of Ir decrease from − 1.601 to − 1.375 e, while the accepting electrons of O increase from 0.799 to 1.121 e (Fig. 5b and Tables S5 and S6). This observation suggests that the valence state of the Ir cations decreases with the addition of Ta, potentially enhancing the resistance of Ir to dissolution in acid environments. Furthermore, the reduction in covalency for Ir–O resulted in the formation of additional Ta–O bonds. The strong electronic interaction in the resulting Ir–O–Ta local structure contributes to the formation of a more stable surface configuration, which may further suppress the involvement of lattice oxygen during OER, thereby improving catalyst stability [63]. Figure 5c depicts the projected density of states (PDOS) of the simulated structures of (Ir, Ta)O_2_ and IrO_2_. The introduction of Ta leads to an increase in the total density of states near the Fermi energy level, indicating that Ta atoms can effectively modulate the *d*-electron characteristics of Ir, thereby optimizing the local coordination of reactive Ir sites. Additionally, it was observed that the energy levels of the orbitals for Ta and O atoms gradually raised, with the strongest hybridization occurring near the Fermi energy level. This observation further supports the preferential formation of Ta-O bonds. Moreover, a downward shift in the *d*-band center is noted for (Ir, Ta)O_2_ (− 2.29 eV) compared to IrO_2_ (− 1.93 eV), which can effectively weaken the adsorption strength of O-based intermediates, thereby enhancing the catalytic efficiency during the acidic OER process [64]. We also calculated the OER free energy diagrams of IrO_2_ and (Ir, Ta)O_2_ at a zero applied potential of U = 0 V (standard potential). The overall OER reaction pathway in acidic media mainly follows the AEM, involving four concerted proton–electron pairs transfer. The rate-determining step (RDS) is identified as the conversion from *O to *OOH for both (Ir, Ta)O_2_ and IrO_2_ (Fig. 5d). The relative energy barrier of the (Ir, Ta)O_2_ model is reduced to 2.277 eV, significantly lower than that of IrO_2_ (2.618 eV), suggesting that Ir sites in (Ir, Ta)O_2_ are efficient active sites to greatly promote reaction kinetics of water oxidation. This enhancement can be primarily attributed to the electronic synergistic effect between Ir and Ta, which optimizes the adsorption strengths of the O-based intermediates by downshifting the *d*-band center, thereby modulating the adsorption energies of the OER intermediates.

## Conclusion

In summary, we have developed a unique IrO_2_/IrTaO_*x*_ bi-layer nanostructure for acidic OER by using a specially designed Ir-Ta-Ni-Nb MG as the matrix. During the OER process, the nanoporous IrO_2_ surface with high catalytical activity was formed in situ on the amorphous IrTaO_*x*_, which provides more accessible active sites and enhances electron transfer. Furthermore, the IrTaO_*x*_ nanostructure beneath can dynamically evolve into IrO_2_ nanocrystals through inward crystallization and dissolution, efficiently replenishing the depletion of surface-active sites. As a result, the self-constructed bi-layered catalyst exhibits a high mass activity of 1.06 A mg_Ir_^−1^ at a 300 mV overpotential and delivers more than 650 h of long-term OER stability under a current density of 100 mA cm^−2^. Electronic structure analyses, combined with DEMS measurements, reveal that the strong electronic interaction between Ir and Ta can balance the valence state of active Ir species, which prevents the formation of hypervalent soluble species and suppresses the involvement of lattice oxygen during OER, thereby further enhancing the catalytic stability. This work provides new insight into the stabilization of active Ir oxides for long-term acidic OER and opens a new avenue for the development of efficient and durable catalysts for practical PEM water electrolysis applications.

## Supplementary Information

Supplementary file 1
