# Supplementary material for: Durable Acidic Oxygen Evolution Via Self-Construction of Iridium Oxide/Iridium-Tantalum Oxide Bi-Layer Nanostructure with Dynamic Replenishment of Active Sites
Source: Nanomicro Lett. 2025 Feb 25;17:165. doi: 10.1007/s40820-025-01680-w (PMC11861462; doi:10.1007/s40820-025-01680-w)
Supplement: Supplementary file 1 [file 40820_2025_1680_MOESM1_ESM.docx]

Supporting Information for

**Durable Acidic Oxygen Evolution via Self-Construction of** **Iridium Oxide/Iridium-Tantalum Oxide** **Bi-Layer Nanostructure with Dynamic Replenishment of Active Sites**

Qi Guo^1^, Rui Li^1,^ *, Yanan Zhang^1^, Qiqin Zhang^1^, Yi He^1^, Zhibin Li^2^, Weihong Liu^3^, Xiongjun Liu^2,^ *, Zhaoping Lu^2,^ *

^1^ Institute of Clean Energy, Yangtze River Delta Research Institute, Northwestern Polytechnical University, Xi'an, 710072, P. R. China

^2^ Beijing Advanced Innovation Center for Materials Genome Engineering, State Key Laboratory for Advanced Metals and Materials, University of Science and Technology Beijing, Beijing 100083, P. R. China

^3^ School of Materials Science and Engineering, Harbin Institute of Technology Shenzhen, Shenzhen, 518055, P. R. China

*Corresponding authors. E-mail: [ruili@nwpu.edu.cn](mailto:ruili@nwpu.edu.cn) (Rui Li); [xjliu@ustb.edu.cn](mailto:xjliu@ustb.edu.cn) (Xiongjun Liu); [luzp@ustb.edu.cn](mailto:luzp@ustb.edu.cn) (Zhaoping Lu)

**Supplementary Figures and Tables**





**Fig. S****1** XRD pattern of the as-spun Ir_30_Ta_35_Ni_29_Nb_6_ MG


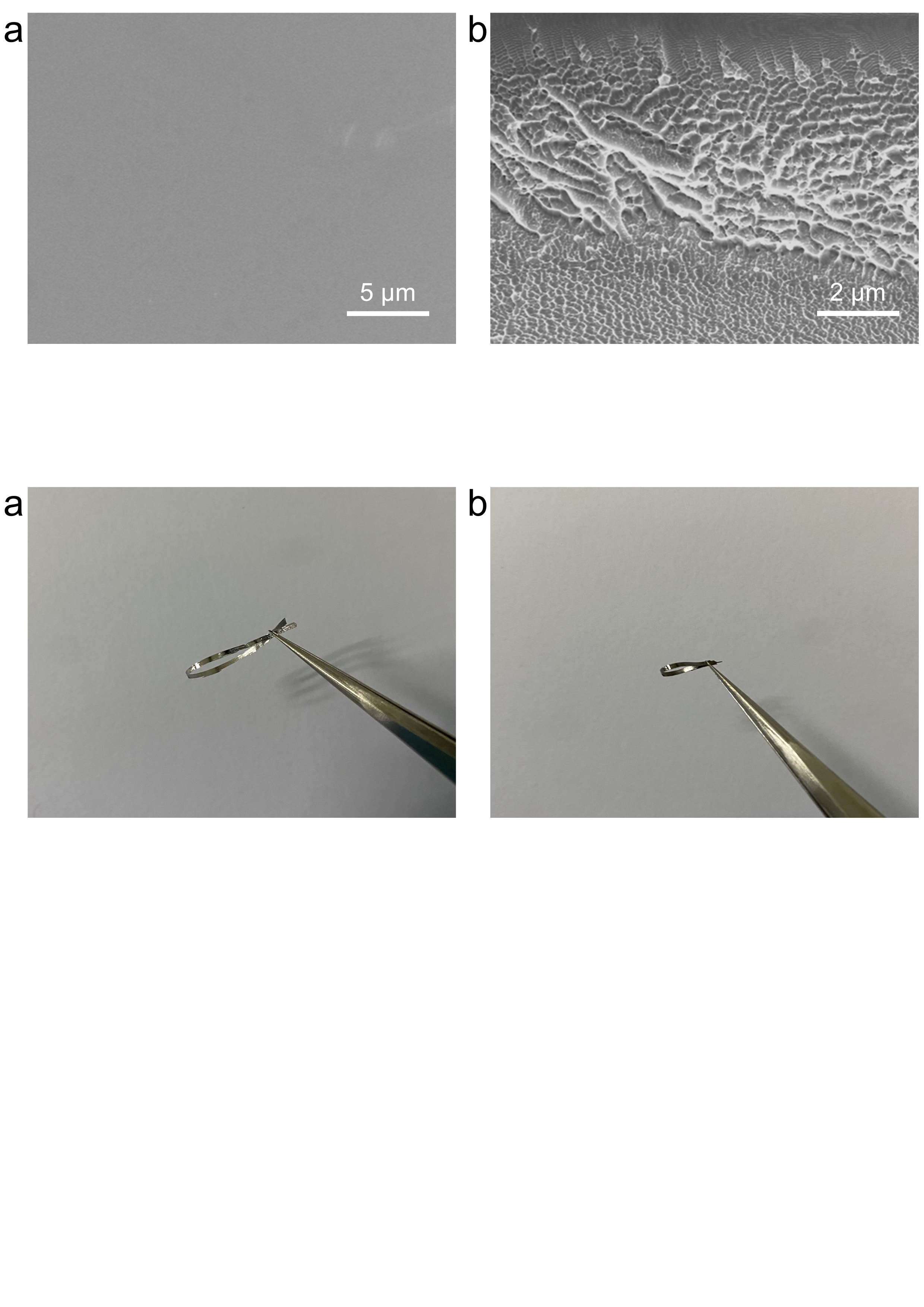


**Fig. S2** The Optical photograph and mechanical flexibility test of (**a**) the as-spun Ir_30_Ta_35_Ni_29_Nb_6_ MG and (**b**) acid-treated Ir_30_Ta_35_Ni_29_Nb_6_ MG


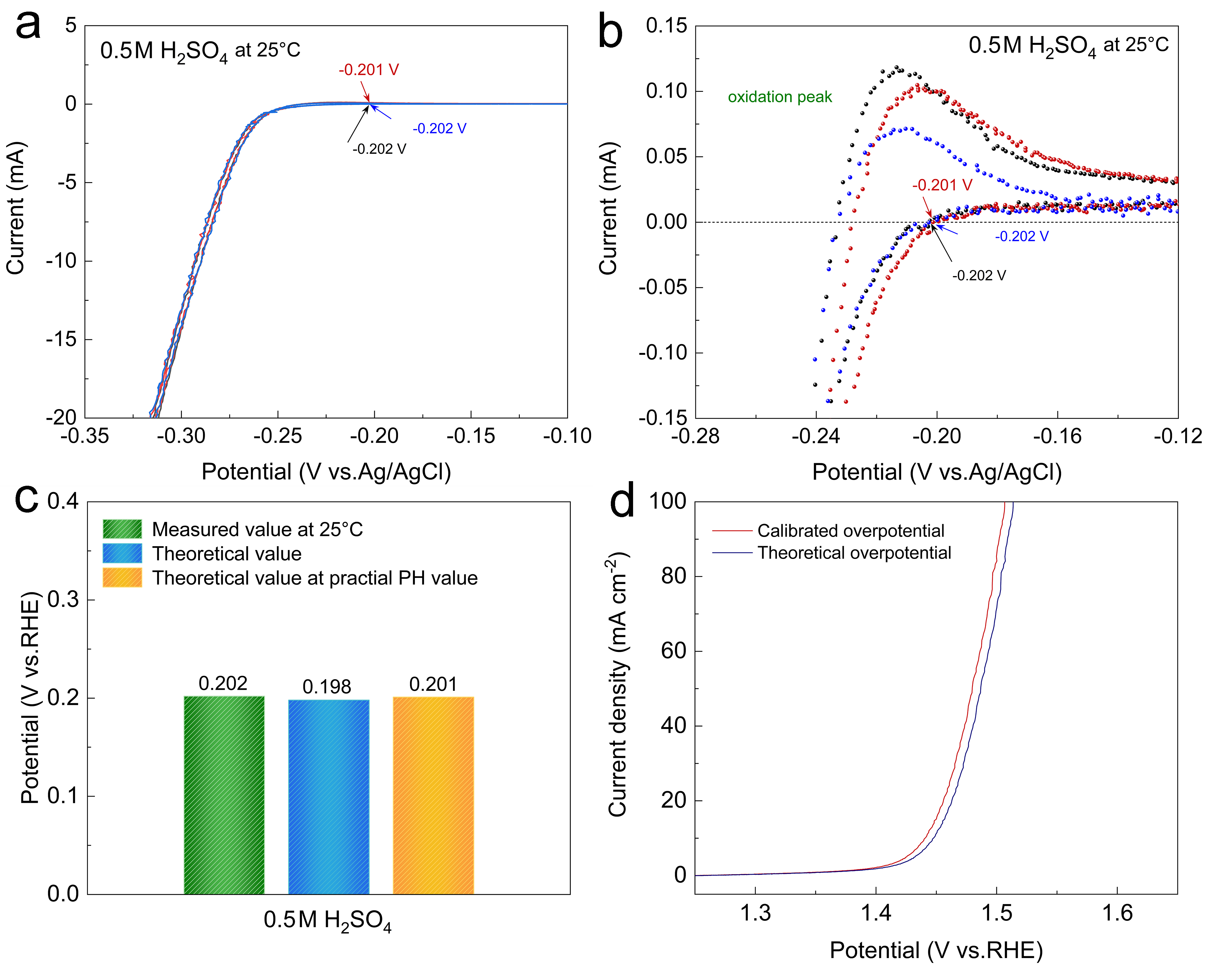


**Fig. S3** (**a**) Single-cycle CV curves of Ag/AgCl electrode calibration in 0.5M H_2_SO_4_ at 25°C. (**b**) The enlarged view of (a). (**c**) RHE calibrated potential of the Ag/AgCl electrode with different methodologies: measured at 25°C (experimental calibration) and theoretically calculated values based on the Nernst equation with pH value in theory and the practical pH value. (**d**) LSV curves of the MG catalyst based on theoretical calculations and experimental corrections





**Fig. S4** OER Nyquist plots of as-spun MG, acid-treated MG, Ir/C, and IrO_2_. The inset shows the enlarged curves in the high-frequency region


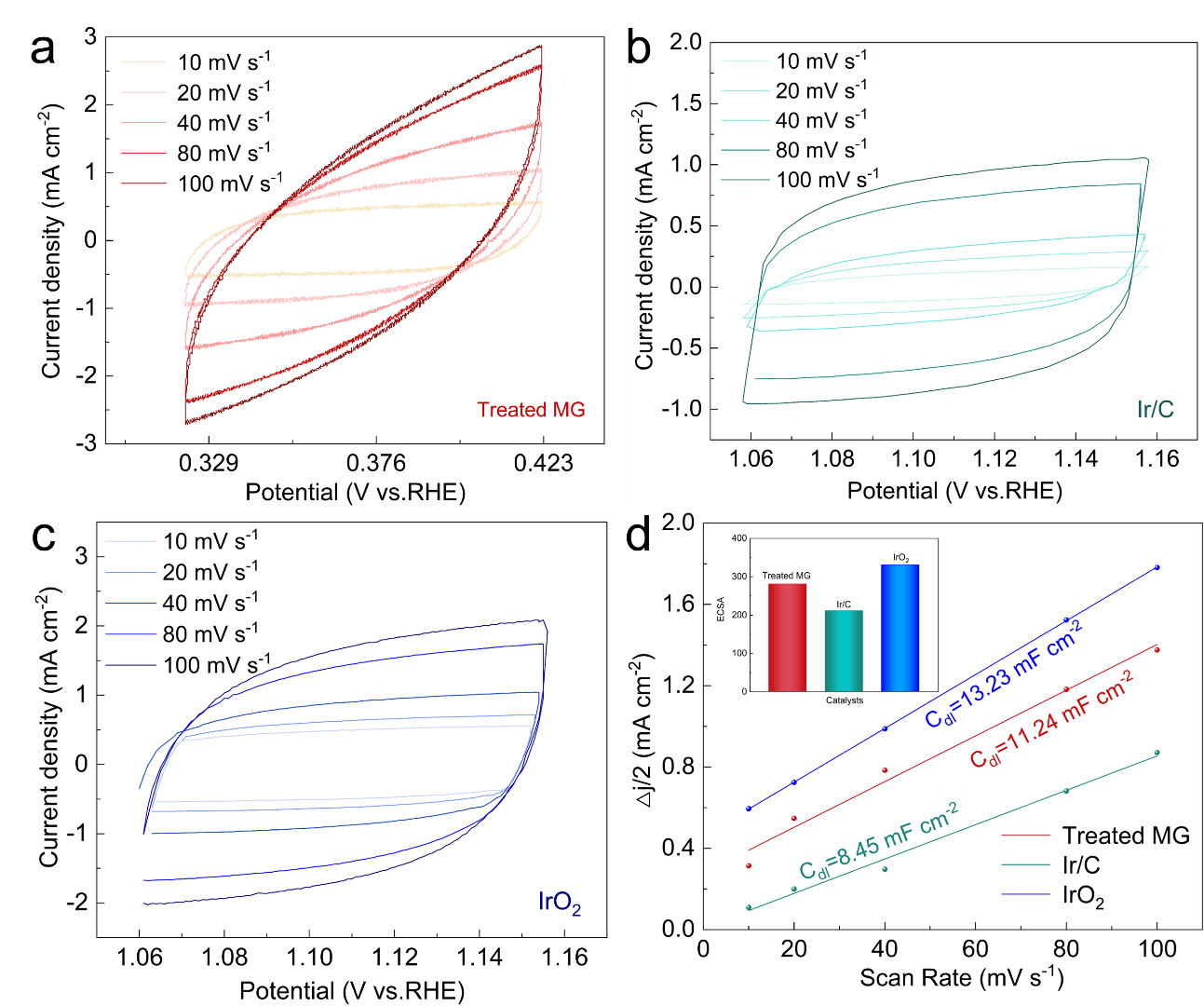


**Fig. S5** Cyclic voltammograms (CVs) of the (a) Treated MG, (b)Ir/C, and (c) IrO_2_ catalysts, respectively. (d) The fitting plots of current densities against scan rates. ∆*j* is the difference between anodic and cathodic current density at a potential of 1.01 V vs RHE. The slope of the fitting line is twice the electrochemical double-layer capacitance, which is proportional to the *ECSA* of the catalysts





**Fig. S6** The *ECSA* normalized current density vs. potential of the catalysts





**Fig. S7** LSV curve of the MG catalyst after the 200-h OER test


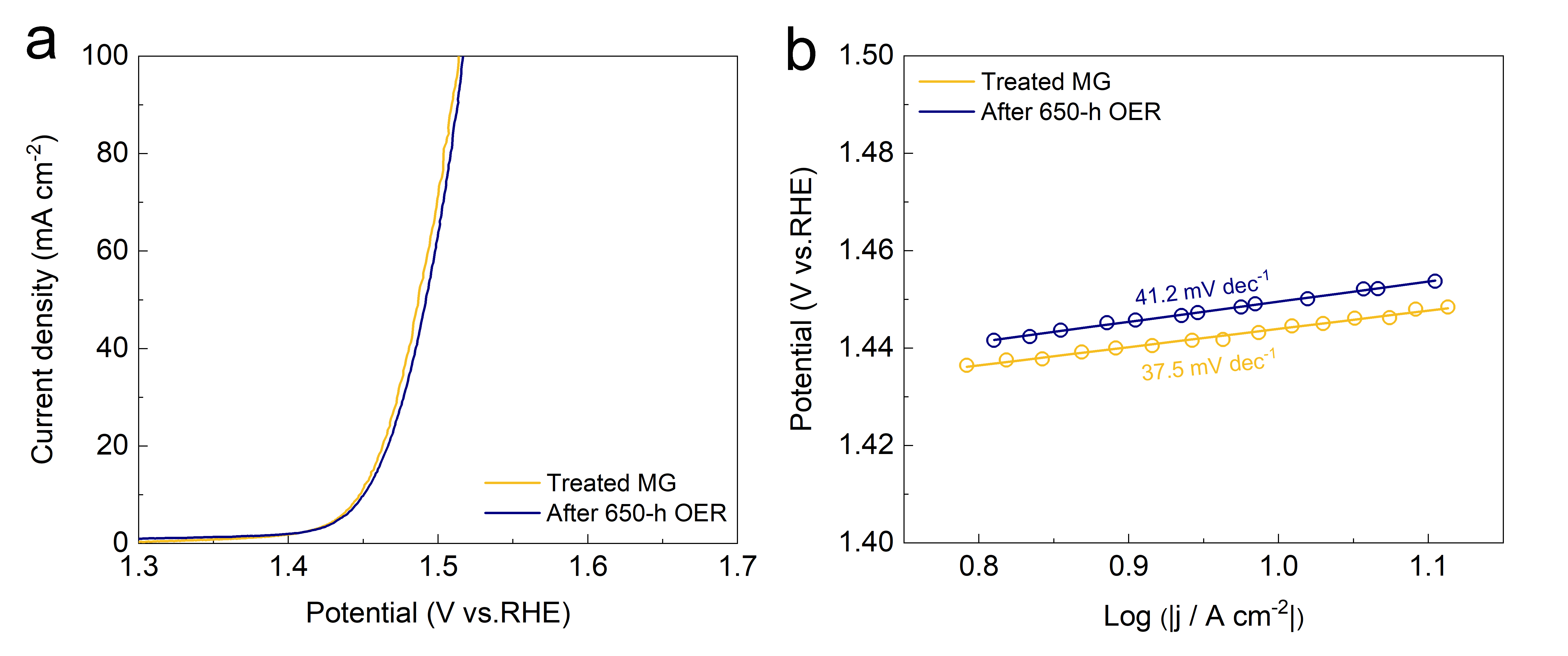
 **Fig. S8** (**a**) Linear polarization curves of the MG catalyst before and after the 650-h OER stability test. (**b**) The corresponding Tafel slopes

**

**

**Fig. S9** Dissolution amount of Ir for the acid-treated MG at 100 mA cm^-2^, the inset is the dissolution amount of Ir for the IrO_2_ and Ir/C at 10 mA cm^-2^

_

_

**Fig. S10** Calculated S-number of the MG catalyst during 650-h OER stability test





**Fig. S11** Chronopotentiometry curve of the MG catalyst at an ampere-level current density of 1 A cm^-2^


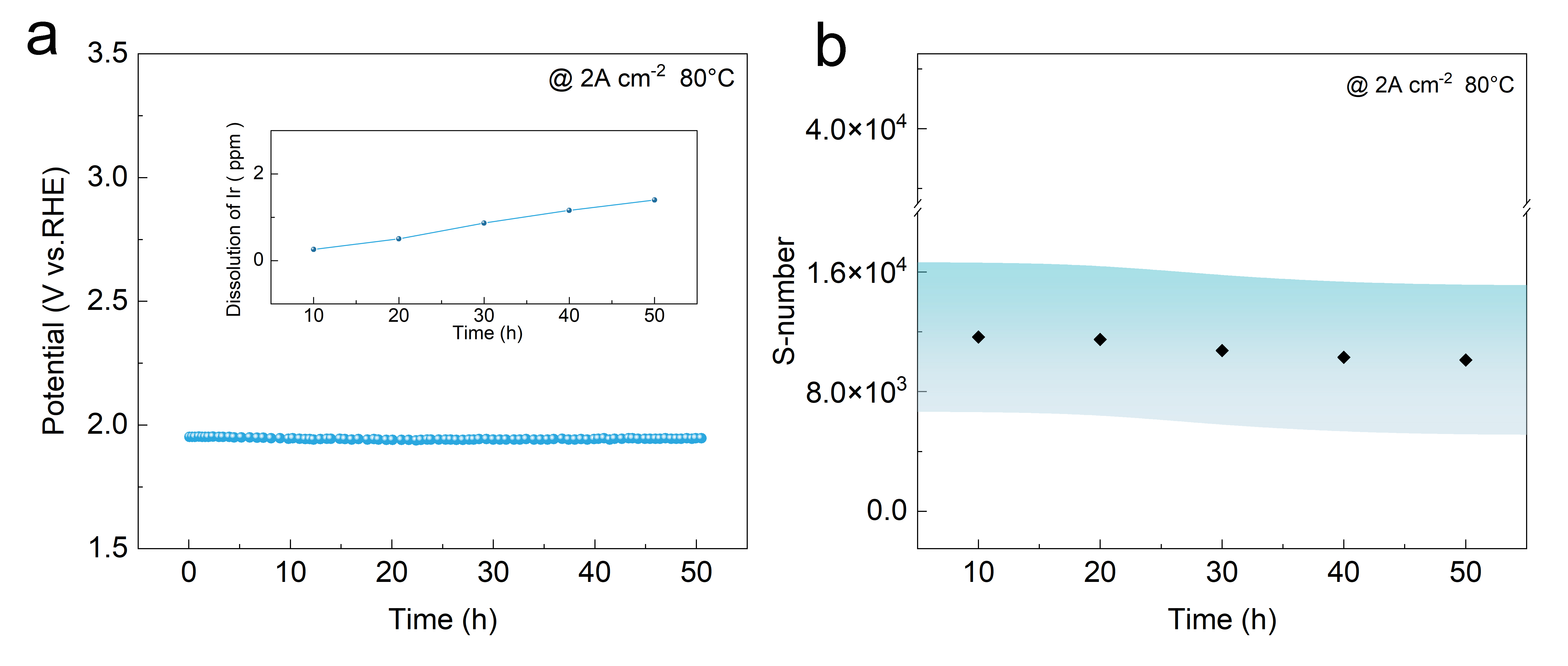


**Fig. S12** (**a**) OER stability test of the MG catalyst under a current density of 2 A cm^-2^ at 80°C. The inset shows the Ir dissolution detected by ICP. (**b**) The calculated S-number during the 50-h OER test


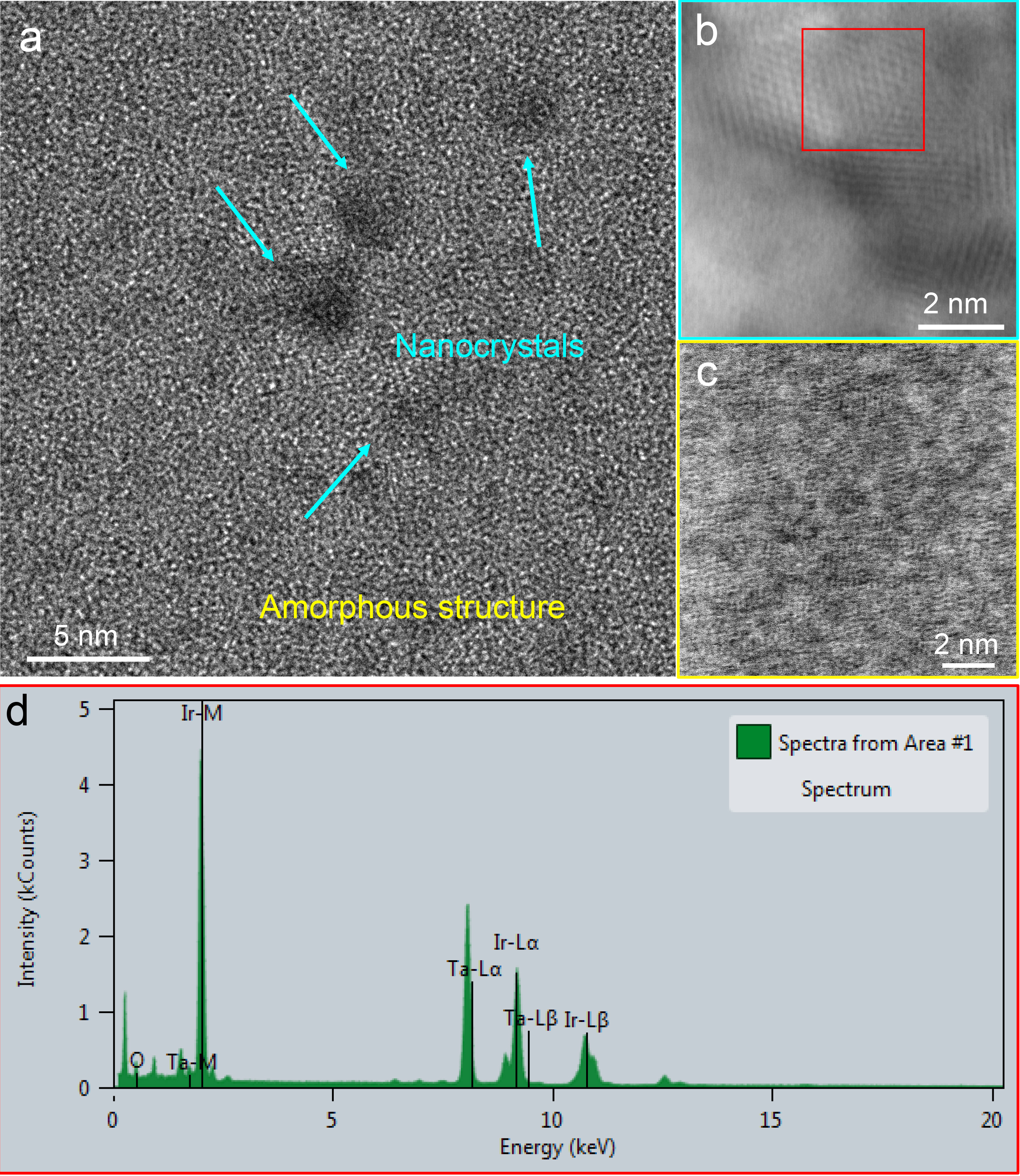


**Fig. S13** (**a**) HRTEM image of the amorphous IrTaO_x_ structure with a few nanocrystals. (**b**) HAADF-STEM image of the nanocrystal. (**c**) HAADF-STEM image of the amorphous structure. (**d**) STEM-EDS analysis of the nanocrystal marked in (b)


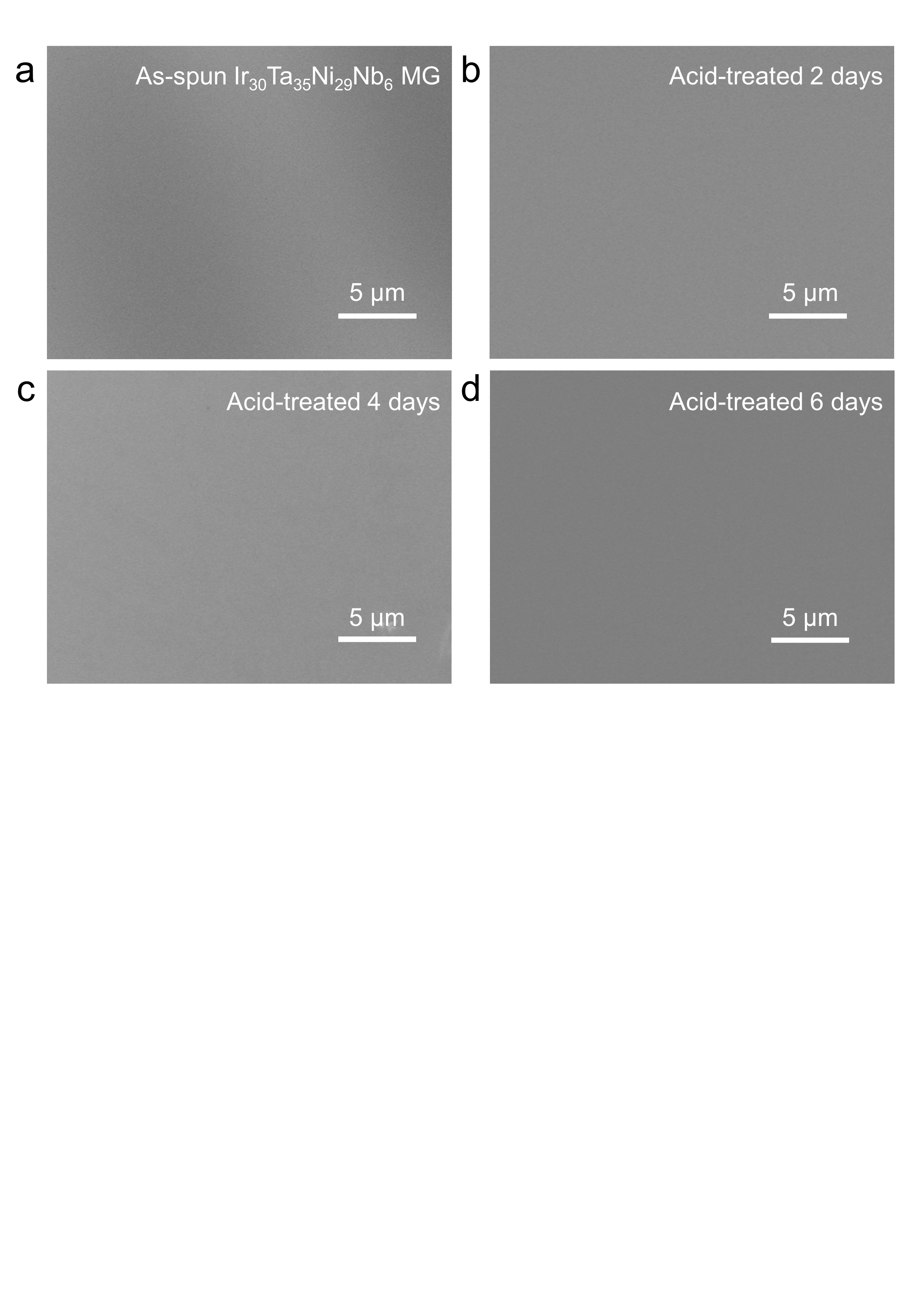


**Fig. S14** Surface SEM morphologies of the as-spun Ir_30_Ta_35_Ni_29_Nb_6_ MG with different leaching durations in HF: (a) initial state; (b) 2 days; (c) 4 days, and (d) 6 days. This result demonstrates the protection of the surface Ta_2_O_5_ passivation layer





**Fig. S15** OER LSV curves of the MG catalysts with different acid treatment times





**Fig. S16** Polarization curve of Ta_2_O_5_ in 0.5 M H_2_SO_4_


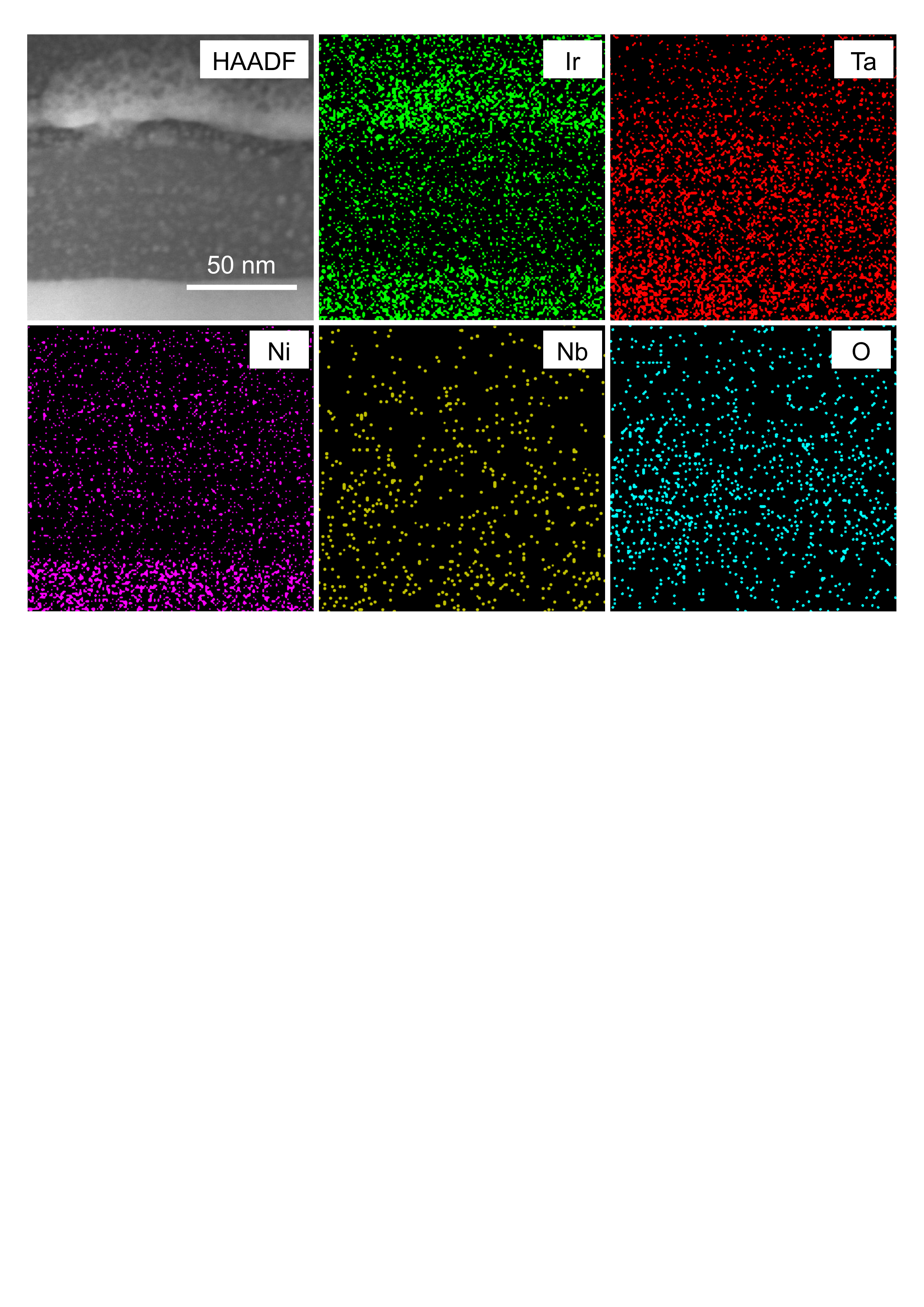


**Fig. S17** HAADF-TEM image and the elemental mapping of the reacted layer structure after 200-h OER


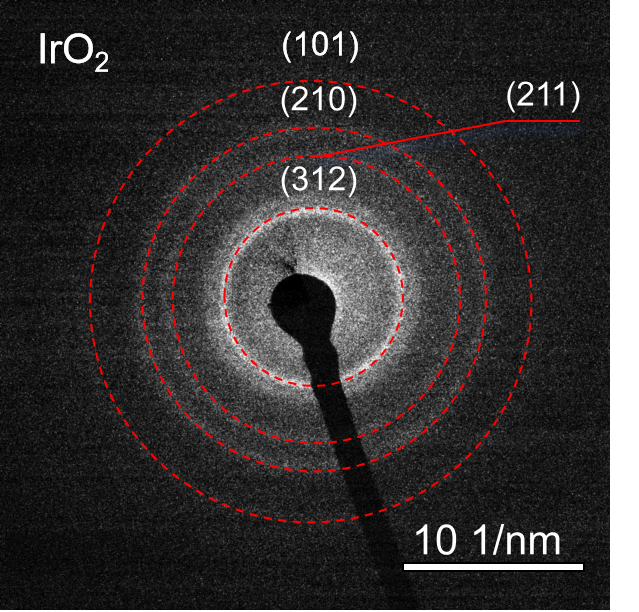


**Fig. S18** SAED pattern of the dispersed IrO_2_ nanocrystal within the underlying IrTaO_x_ structure
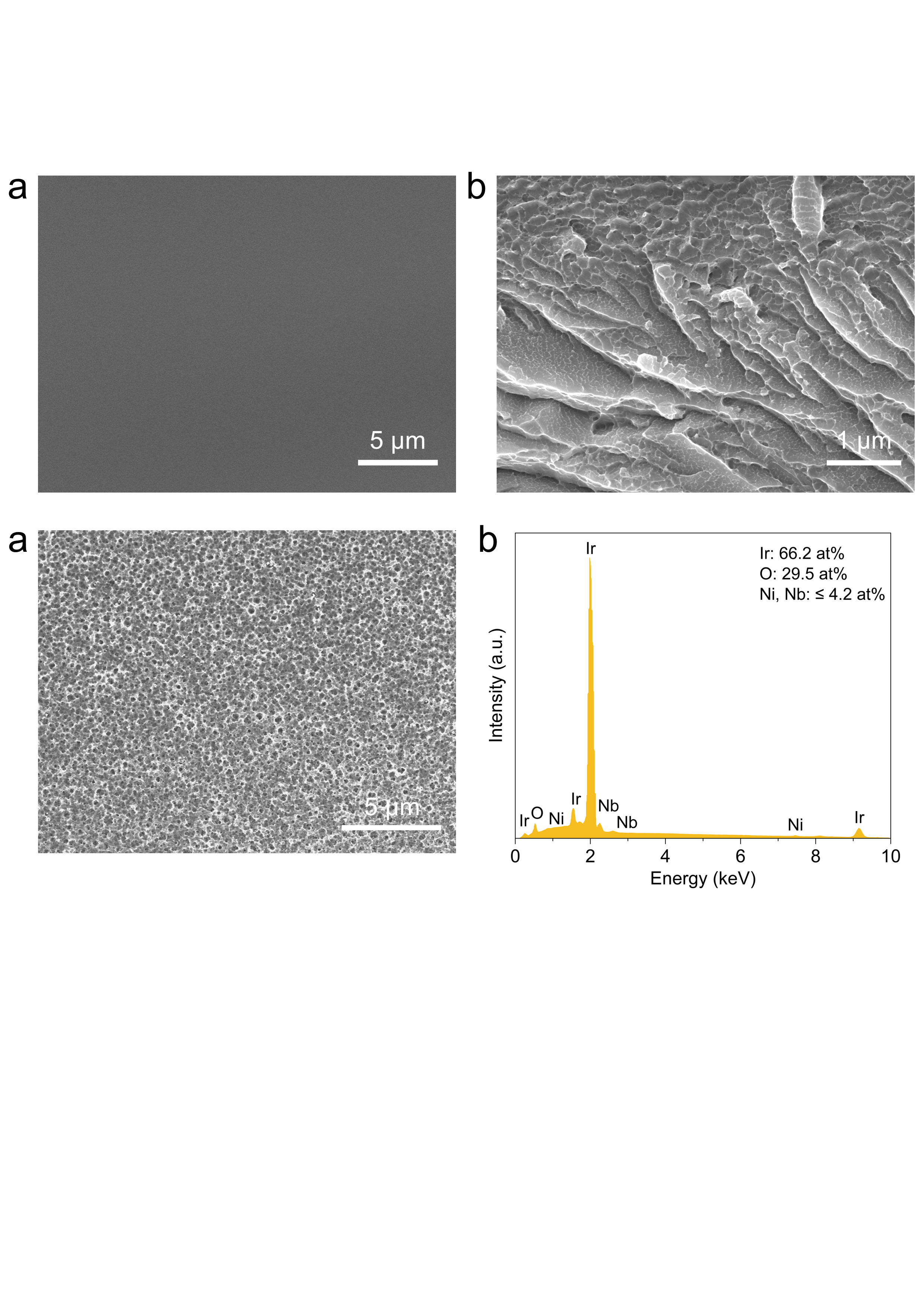


**Fig. S19** (**a**) Surface and (**b**) cross-sectional SEM image of the as-spun Ir_30_Ni_42_Nb_28_ MG with an atomic ratio of 30:42:28





**Fig. S20** TEM-EDS analysis of the as-spun Ir_30_Ni_42_Nb_28_ MG


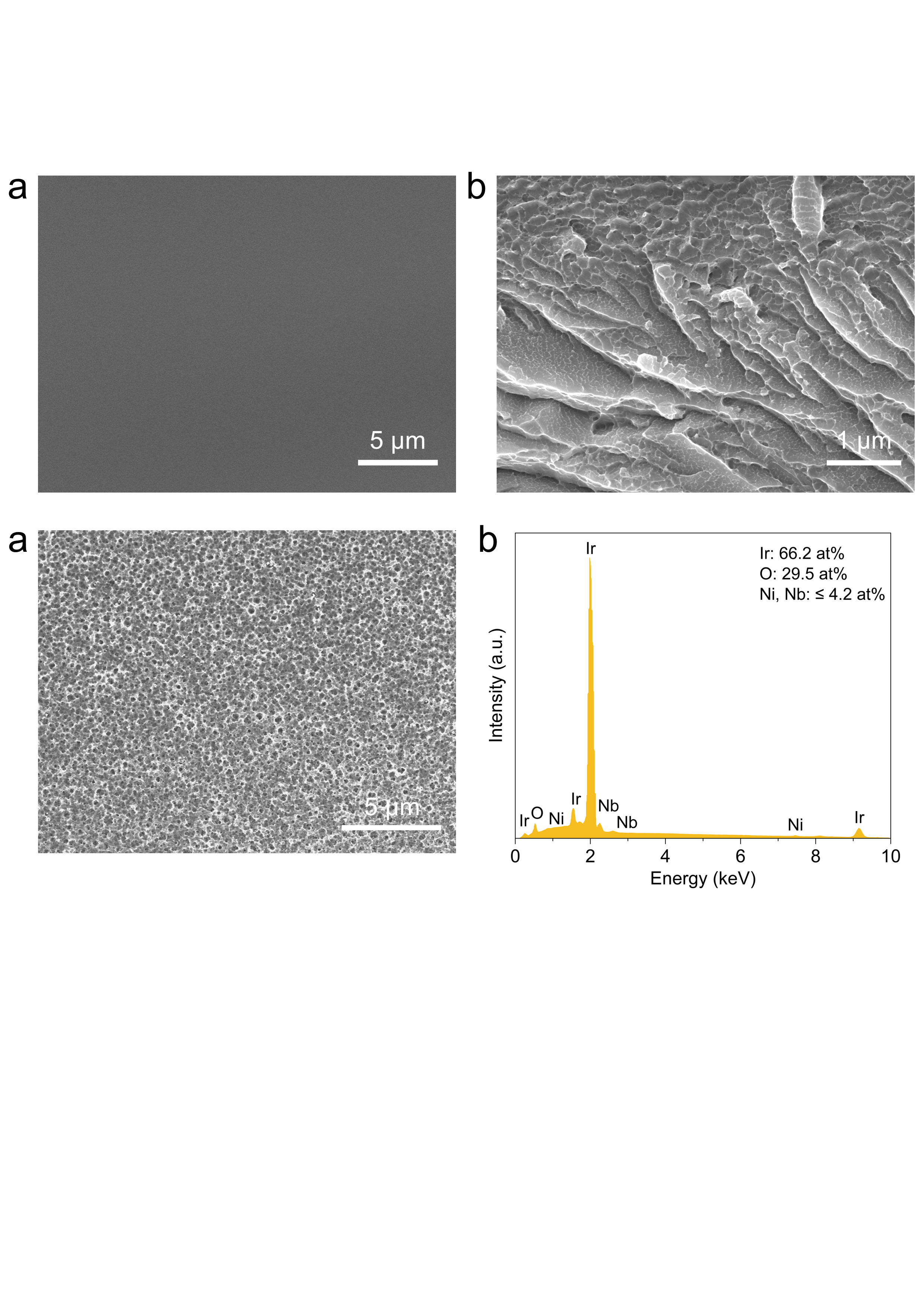


**Fig. S21** (**a**) Surface SEM morphology and (**b**) EDS analysis of the acid-treated Ir_30_Ni_42_Nb_28_ MG





**Fig. S22** OER Linear polarization curves of the acid-treated Ir_30_Ta_35_Ni_29_Nb_6_ and Ir_30_Ni_42_Nb_28_ MG catalysts in a 0.5 M H_2_SO_4_ electrolyte





**Fig. S23** Chronopotentiometry curve of the treated Ir_30_Ni_42_Nb_28_ MG catalyst at a current density of 100 mA cm^-2^





**Fig. S24** Dissolution amount of Ir for the treated Ir_30_Ni_42_Nb_28_ MG during the 80 h at 100 mA cm^-2^


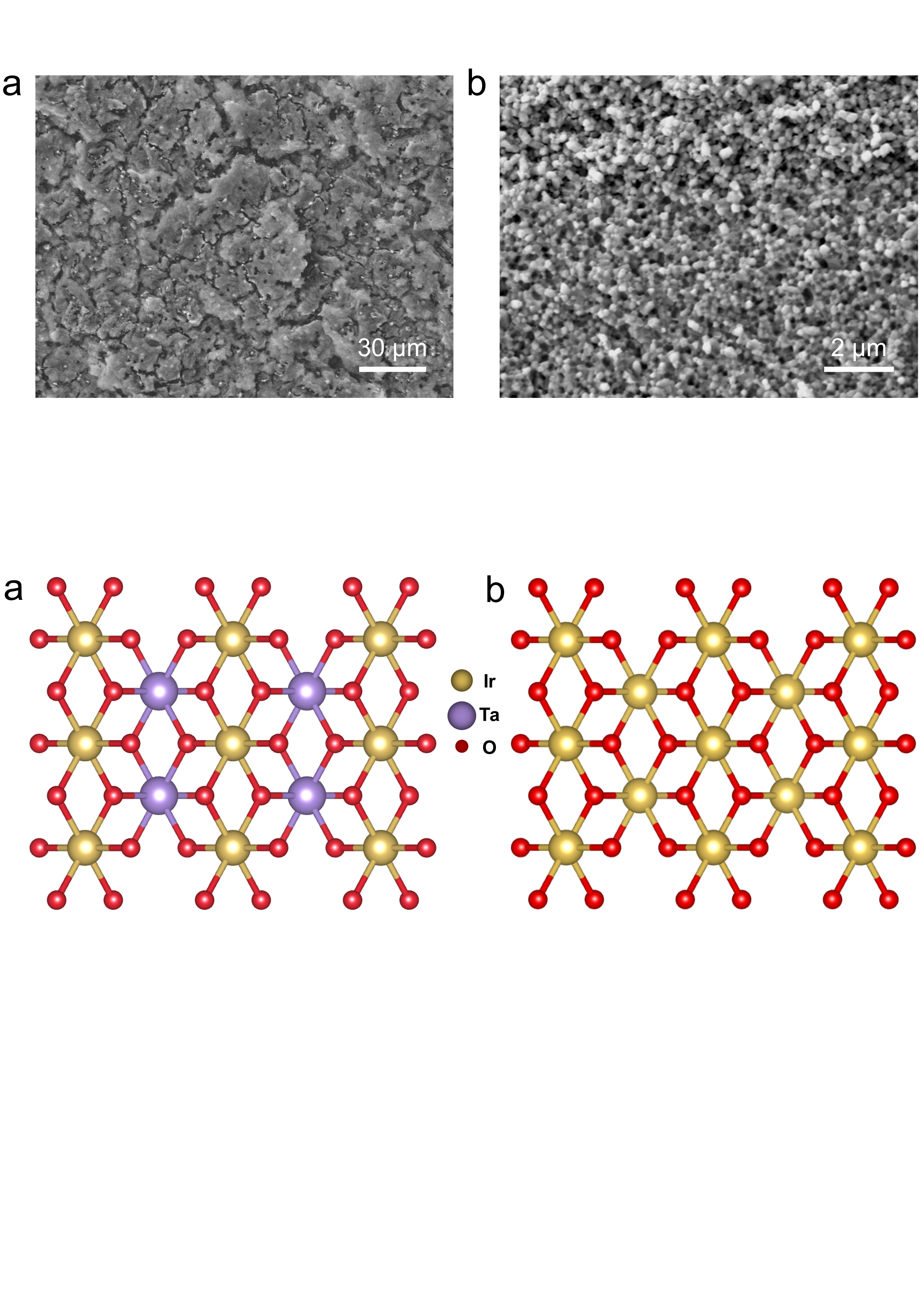


**Fig. S25** SEM images of the acid-treated Ir_30_Ni_42_Nb_28_ MG after the OER stability test: (**a**) Surface; (**b**) Cross section


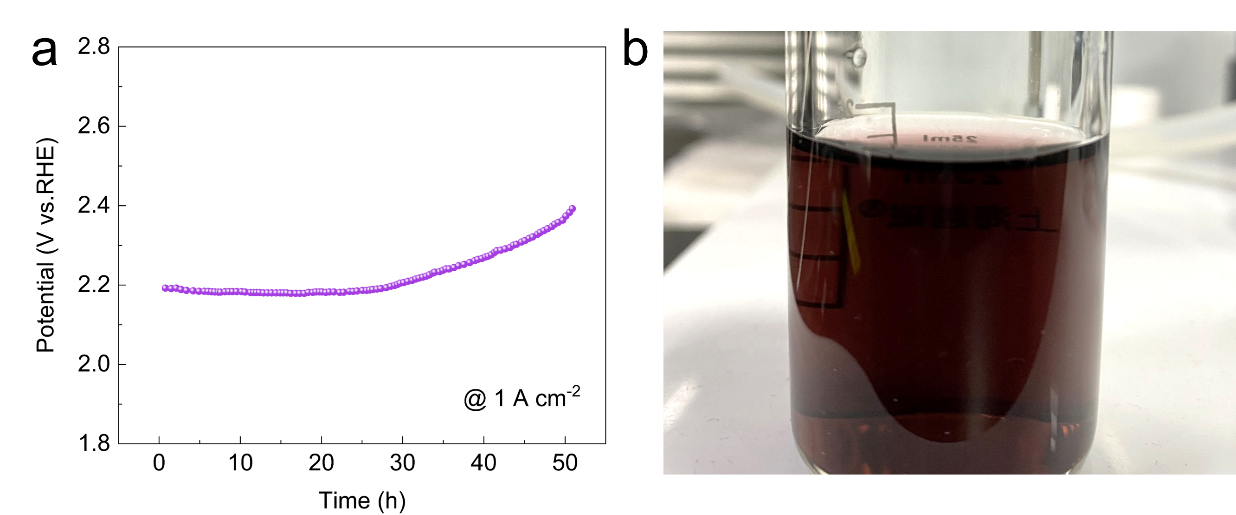


**Fig. S26** (**a**) Chronopotentiometry curve of the acid-treated Ir_65_Ni_29_Nb_6_ at a current density of 1 A cm^-2^. (**b**) Electrolyte color change after the 50-h OER test


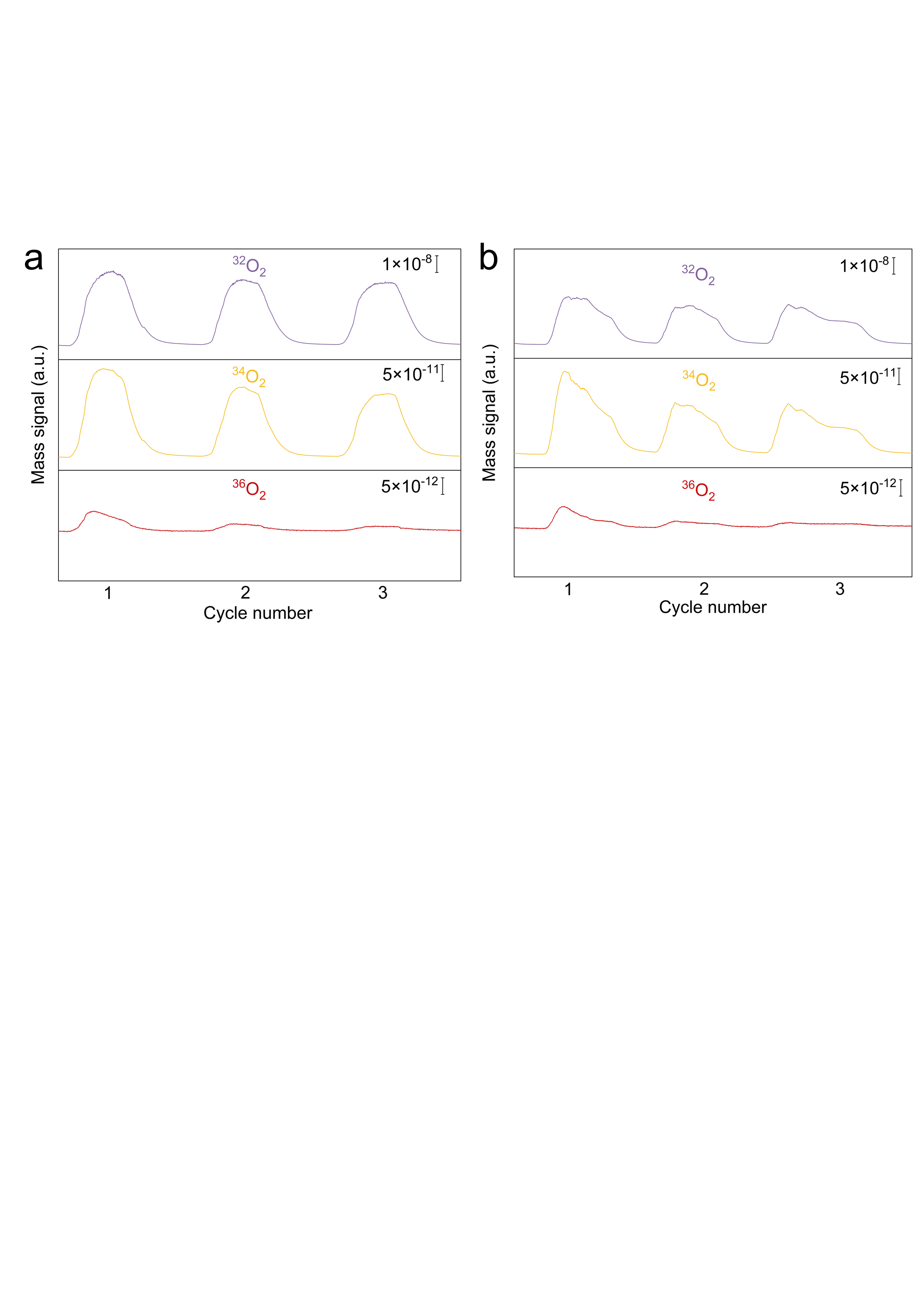


**Fig. S27** DEMS signals of ^32^O_2_ (^16^O^16^O, mass/charge ratio (m/z) = 32), ^34^O_2_(^16^O^18^O, m/z = 34), and ^36^O_2_ (^18^O^18^O, m/z = 36) from the gaseous products for ^18^O-labeled catalysts in H_2_^16^O aqueous H_2_SO_4_ electrolyte during 3 cycles: (**a**) treated Ir_30_Ta_35_Ni_29_Nb_6_ MG, (**b**) treated Ir_30_Ni_42_Nb_28_ MG


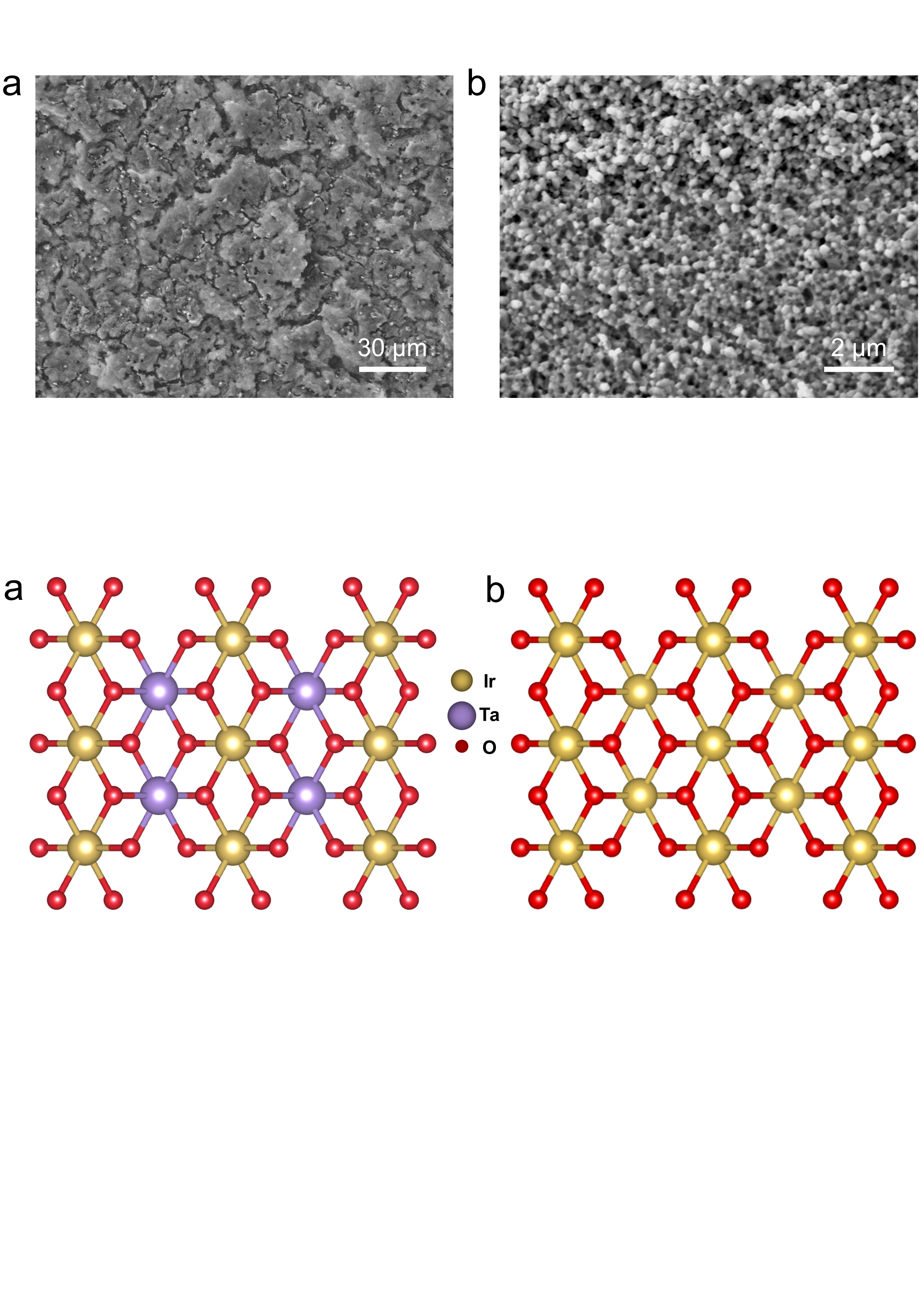


**Fig. S28** The (110) crystal plane for (a) (Ir, Ta)O_2_ model and (b) pristine IrO_2_ model

**Table S1** Overpotential and Tafel slope comparison of the treated Ir_30_Ta_35_Ni_29_Nb_6_ MG catalyst with previously reported Ir-based catalysts in acidic media.

| *Catalysts* | *Electrolyte* | *Overpotential (mV)* | *Tafel slope*  *(mV dec^-1^)* | *Stability*  *(h @ mA cm^-2^)* | *Ref* |
| --- | --- | --- | --- | --- | --- |
| Treated Ir_30_Ta_35_Ni_29_Nb_6_ MG | 0.5 M H_2_SO_4_ | η_10_ = 211 mV (218 mV corrected) | 37.52 | 650 h@100 mA cm^-2^ | This work |
| Ir/Nb_2_O_5-x_ | 0.5 M H_2_SO_4_ | η_10_ = 218 mV | 52.3 | 105 h@10 mA cm^-2^ | [S1] |
| np-WIr | 0.5 M H_2_SO_4_ | η_10_ = 291 mV | 78 | 800 h@100 mA cm^-2^ | [S2] |
| Ir_1_Ru_4_/TiC | 0.1 M HClO_4_ | η_10_ = 230 mV | / | 20 h@10 mA cm^-2^ | [S3] |
| HEA@Ir-MEO | 0.5 M H_2_SO_4_ | η_10_ = 243 mV | 56.2 | 24 h@50 mA cm^-2^ | [S4] |
| Ir-Co_3_O_4_ | 0.5 M H_2_SO_4_ | η_10_ = 236 mV | 52.6 | 30 h@10 mA cm^-2^ | [S5] |
| ZnNiCoIrMn | 0.1 M HClO_4_ | η_10_ = 237 mV | 46 | 100 h@10 mA cm^-2^ | [S6] |
| Ta_0.1_Tm_0.1_Ir_0.8_O_2-x_ | 0.5 M H_2_SO_4_ | η_10_ = 296 mV | 64 | 500 h@10 mA cm^-2^ | [S7] |
| Au@AuIr_2_ | 0.5 M H_2_SO_4_ | η_10_ = 261 mV | 58.3 | 40 h@10 mA cm^-2^ | [S8] |
| CeO_2_@SrIrO_3_ | 0.5 M H_2_SO_4_ | η_10_ = 238 mV | 71.7 | 50 h@10 mA cm^-2^ | [S9] |
| Ti-IrO_x_/Ir | 0.5 M H_2_SO_4_ | η_10_ = 254 mV | 48 | 100 h@10 mA cm^-2^ | [S10] |
| RuIrAl | 0.5 M H_2_SO_4_ | η_10_ = 178 mV | 49.2 | 300 h@100 mA cm^-2^ | [S11] |
| Ir-W@Ir-WO_3-x_ | 0.5 M H_2_SO_4_ | η_10_ = 261 mV | 65 | 20 h@10 mA cm^-2^ | [S12] |
| Ir nanosheets | 0.5 M H_2_SO_4_ | η_10_ = 254 mV | 72.5 | 50 h@10 mA cm^-2^ | [S13] |
| PdCu/Ir/C | 0.1 M HClO_4_ | η_10_ = 283 mV | 59.6 | 15 h@10 mA cm^-2^ | [S14] |
| Ir-Sn PSC | 0.5 M H_2_SO_4_ | η_10_ = 225 mV | 64.1 | 180 h@30 mA cm^-2^ | [S15] |
| Ir_0.06_Co_2.94_O_4_ | 0.1 M HClO_4_ | η_5_ = 300 mV | 45 | 200 h@10 mA cm^-2^ | [S16] |
| IrTe nanotubes | 0.5 M H_2_SO_4_ | η_10_ = 271 mV | 44.65 | 24 h@10 mA cm^-2^ | [S17] |
| c-IrO_x_-MoO_3_/Ti | 0.5 M H_2_SO_4_ | η_10_ = 200 mV | 58.6 | 130 h@100 mA cm^-2^ | [S18] |
| IrHf_x_O_y_ | 0.1 M HClO_4_ | η_10_ = 330 mV | 60 | / | [S19] |
| Re_0.1_-IrO_2_ | 0.5 M H_2_SO_4_ | η_10_ = 255m V | 65.6 | 170 h@10 mA cm^-2^ | [S20] |

**Table S2** Mass activity comparison of the treated Ir_30_Ta_35_Ni_29_Nb_6_ MG catalyst with previously reported Ir-based catalysts in acidic media.

| *Catalyst* | *Electrolyte* | *Mass activity* | *Ref* |
| --- | --- | --- | --- |
| Treated Ir_30_Ta_35_Ni_29_Nb_6_ MG | 0.5 M H_2_SO_4_ | 1.06 A mg_Ir_^-1^@300 mV | This work |
| IrGa-IMC@IrO_x_ | 0.1 _M_ HClO_4_ | 0.841A mg_Ir_^-1^@290 mV | [S21] |
| Sm_3_IrO_7_ | 0.1 M HClO_4_ | 0.307A mg_Ir_^-1^@300 mV | [S22] |
| DO-IrTe_2_ HNSs | 0.5 M H_2_SO_4_ | 0.214A mg_Ir_^-1^@300 mV | [S23] |
| Rh_22_Ir_78_/VXC | 0.5 M H_2_SO_4_ | 1.170A mg_Ir_^-1^@300 mV | [S24] |
| IrNi-RF | 0.1 M HClO_4_ | 0.470A mg_Ir_^-1^@300 mV | [S25] |
| Ru_1_Ir_1_O_x_ | 0.5 M H_2_SO_4_ | 1.124A mg_Ir_^-1^@300 mV | [S26] |
| IrCoNi | 0.1 M HClO_4_ | 0.720A mg_Ir_^-1^ @300 mV | [S27] |
| PtIrNi | 0.5 M H_2_SO_4_ | 0.650A mg_Ir_^-1^@300 mV | [S28] |
| dotf-IrCo_5_ | 0.1 M HClO_4_ | 0.165A mg_Ir_^-1^@250 mV | [S29] |
| Ir-NSs | 0.5 M H_2_SO_4_ | 0.209A mg_Ir_^-1^@300 mV | [S30] |
| 22 wt% Ir/δ-MnO_2_ | 0.5 M H_2_SO_4_ | 0.140A mg_Ir_^-1^@300 mV | [S31] |
| Li-IrSe_2_ | 0.5 M H_2_SO_4_ | 0.066A mg_Ir_^-1^@220 mV | [S32] |
| IrNiO_x_ | 0.5 M H_2_SO_4_ | 0.676A mg_Ir_^-1^@300 mV | [S33] |

**Table S3** ICP-detected surface composition of the treated Ir_30_Ta_35_Ni_29_Nb_6_ MG catalyst

| *Elements* | *At%* |
| --- | --- |
| Ir | 48.48% |
| Ta | 50.94% |
| Ni | 0.00% |
| Nb | 0.58% |

**Table S4** SEM-EDS elemental compositions of the Ir_30_Ni_42_Nb_28_ MG catalyst after the acidic OER stability test

| *Sample* | *Atomic ratio (%)* | | | |
| --- | --- | --- | --- | --- |
|  | *Ir* | *Ni* | *Nb* | *O* |
| Treated Ir_30_Ni_42_Nb_28_ MG (after test) | 7.36 | 15.70 | 4.79 | 72.15 |

**Table S5** Calculated electron transfer in (Ir, Ta)O_2_

| *Sites* | *Electrons* | *Valence electron* | *Electronic transfer* |
| --- | --- | --- | --- |
| Ta1 | 8.099072 | 11 | -2.900928 |
| Ta2 | 8.099072 | 11 | -2.900928 |
| Ta3 | 8.099072 | 11 | -2.900928 |
| Ta4 | 8.099072 | 11 | -2.900928 |
| Ta5 | 8.099072 | 11 | -2.900928 |
| Ta6 | 8.099072 | 11 | -2.900928 |
| Ta7 | 8.099072 | 11 | -2.900928 |
| Ta8 | 8.099072 | 11 | -2.900928 |
| Ir1 | 7.619728 | 9 | -1.38027 |
| Ir2 | 7.619728 | 9 | -1.38027 |
| Ir3 | 7.619728 | 9 | -1.38027 |
| Ir4 | 7.619728 | 9 | -1.38027 |
| Ir5 | 7.624175 | 9 | -1.37583 |
| Ir6 | 7.624175 | 9 | -1.37583 |
| Ir7 | 7.624175 | 9 | -1.37583 |
| Ir8 | 7.624175 | 9 | -1.37583 |
| O1 | 7.120526 | 6 | 1.120526 |
| O2 | 7.015631 | 6 | 1.015631 |
| O3 | 7.12719 | 6 | 1.12719 |
| O4 | 7.014957 | 6 | 1.014957 |
| O5 | 7.120526 | 6 | 1.120526 |
| O6 | 7.016305 | 6 | 1.016305 |
| O7 | 7.12719 | 6 | 1.12719 |
| O8 | 7.015631 | 6 | 1.015631 |
| O9 | 7.120526 | 6 | 1.120526 |
| O10 | 7.015631 | 6 | 1.015631 |
| O11 | 7.12719 | 6 | 1.12719 |
| O12 | 7.014957 | 6 | 1.014957 |
| O13 | 7.120526 | 6 | 1.120526 |
| O14 | 7.016305 | 6 | 1.016305 |
| O15 | 7.12719 | 6 | 1.12719 |
| O16 | 7.015631 | 6 | 1.015631 |
| O17 | 7.120526 | 6 | 1.120526 |
| O18 | 7.015631 | 6 | 1.015631 |
| O19 | 7.12719 | 6 | 1.12719 |
| O20 | 7.014957 | 6 | 1.014957 |
| O21 | 7.120526 | 6 | 1.120526 |
| O22 | 7.016305 | 6 | 1.016305 |
| O23 | 7.12719 | 6 | 1.12719 |
| O24 | 7.015631 | 6 | 1.015631 |
| O25 | 7.120526 | 6 | 1.120526 |
| O26 | 7.015631 | 6 | 1.015631 |
| O27 | 7.12719 | 6 | 1.12719 |
| O28 | 7.014957 | 6 | 1.014957 |
| O29 | 7.120526 | 6 | 1.120526 |
| O30 | 7.016305 | 6 | 1.016305 |
| O31 | 7.12719 | 6 | 1.12719 |
| O32 | 7.015631 | 6 | 1.015631 |

**Table S6** Calculated electron transfer in IrO_2_

| *Site* | *Electrons* | *Valence electron* | *Electronic transfer* |
| --- | --- | --- | --- |
| Ir1 | 7.399196 | 9 | -1.600804 |
| Ir2 | 7.399196 | 9 | -1.600804 |
| Ir3 | 7.399196 | 9 | -1.600804 |
| Ir4 | 7.399196 | 9 | -1.600804 |
| Ir5 | 7.399196 | 9 | -1.600804 |
| Ir6 | 7.399196 | 9 | -1.600804 |
| Ir7 | 7.399196 | 9 | -1.600804 |
| Ir8 | 7.399196 | 9 | -1.600804 |
| Ir9 | 7.403614 | 9 | -1.596386 |
| Ir10 | 7.403614 | 9 | -1.596386 |
| Ir11 | 7.403614 | 9 | -1.596386 |
| Ir12 | 7.403614 | 9 | -1.596386 |
| Ir13 | 7.403614 | 9 | -1.596386 |
| Ir14 | 7.403614 | 9 | -1.596386 |
| Ir15 | 7.403614 | 9 | -1.596386 |
| Ir16 | 7.403614 | 9 | -1.596386 |
| O1 | 6.799274 | 6 | 0.799274 |
| O2 | 6.799297 | 6 | 0.799297 |
| O3 | 6.799729 | 6 | 0.799729 |
| O4 | 6.799798 | 6 | 0.799798 |
| O5 | 6.79882 | 6 | 0.79882 |
| O6 | 6.798843 | 6 | 0.798843 |
| O7 | 6.799274 | 6 | 0.799274 |
| O8 | 6.799343 | 6 | 0.799343 |
| O9 | 6.799274 | 6 | 0.799274 |
| O10 | 6.799297 | 6 | 0.799297 |
| O11 | 6.799729 | 6 | 0.799729 |
| O12 | 6.799798 | 6 | 0.799798 |
| O13 | 6.79882 | 6 | 0.79882 |
| O14 | 6.798843 | 6 | 0.798843 |
| O15 | 6.799274 | 6 | 0.799274 |
| O16 | 6.799343 | 6 | 0.799343 |
| O17 | 6.799274 | 6 | 0.799274 |
| O18 | 6.799297 | 6 | 0.799297 |
| O19 | 6.799729 | 6 | 0.799729 |
| O20 | 6.799798 | 6 | 0.799798 |
| O21 | 6.79882 | 6 | 0.79882 |
| AO22 | 6.798843 | 6 | 0.798843 |
| O23 | 6.799274 | 6 | 0.799274 |
| O24 | 6.799343 | 6 | 0.799343 |
| O25 | 6.799274 | 6 | 0.799274 |
| O26 | 6.799297 | 6 | 0.799297 |
| O27 | 6.799729 | 6 | 0.799729 |
| O28 | 6.799798 | 6 | 0.799798 |
| O29 | 6.79882 | 6 | 0.79882 |
| O30 | 6.798843 | 6 | 0.798843 |
| O31 | 6.799274 | 6 | 0.799274 |
| O32 | 6.799343 | 6 | 0.799343 |

**Supplementary References**

1. Z. Shi, J. Li, J. Jiang, Y. Wang, X. Wang et al., Enhanced acidic water oxidation by dynamic migration of oxygen species at the Ir/Nb_2_O_5-x_ catalyst/support interfaces. Angew Chem Int Ed Engl. **61**, e202212341 (2022). <https://doi.org/10.1002/anie.202212341>
2. R. Li, H. Wang, F. Hu, K. C. Chan, X. Liu et al., IrW nanochannel support enabling ultrastable electrocatalytic oxygen evolution at 2 A cm^-2^ in acidic media. Nat Commun. **12**, 3540 (2021). <https://doi.org/10.1038/s41467-021-23907-1>
3. J. Zhang, X. Cao, Y. F. Jiang, S. F. Hung, W. Liu et al., Surface enrichment of Ir on the IrRu alloy for efficient and stable water oxidation catalysis in acid. Chem. ci. **13**, 12114-12121 (2022). <https://doi.org/10.1039/d2sc03947h>
4. L. Yao, F. Zhang, S. Yang, H. Zhang, Y. Li et al., Sub-2 nm IrRuNiMoCo high-entropy alloy with iridium-rich medium-entropy oxide shell to boost acidic oxygen evolution. Adv. ater. **36**, 2314049 (2024). <https://doi.org/10.1002/adma.202314049>
5. Y. Zhu, J. Wang, T. Koketsu, M. Kroschel, J. M. Chen et al., Iridium single atoms incorporated in Co_3_O_4_ efficiently catalyze the oxygen evolution in acidic conditions. Nat. ommun. **13**, 7754 (2022). <https://doi.org/10.1038/s41467-022-35426-8>
6. J. Kwon, S. Sun, S. Choi, K. Lee, S. Jo et al., Tailored electronic structure of Ir in high entropy alloy for highly active and durable bifunctional electrocatalyst for water splitting under an acidic environment. Adv. ater. **35**, 2300091 (2023). <https://doi.org/10.1002/adma.202300091>
7. S. Hao, H. Sheng, M. Liu, J. Huang, G. Zheng et al., Torsion strained iridium oxide for efficient acidic water oxidation in proton exchange membrane electrolyzers. Nat Nanotechnol. **16**, 1371-1377 (2021). <https://doi.org/10.1038/s41565-021-00986-1>
8. H. Wang, Z. N. Chen, D. Wu, M. Cao, F. Sun et al., Significantly enhanced overall water splitting performance by partial oxidation of Ir through au modification in core-shell alloy structure. J. Am. Chem. Soc. **143**, 4639-4645 (2021). <https://doi.org/10.1021/jacs.0c12740>
9. M. You, Y. Xu, B. He, J. Zhang, L. Gui et al., Realizing robust and efficient acidic oxygen evolution by electronic modulation of 0D/2D CeO_2_ quantum dots decorated SrIrO_3_ nanosheets. Appl Catal. **315**, 121579 (2022). <https://doi.org/10.1016/j.apcatb.2022.121579>
10. Y. Wang, R. Ma, Z. Shi, H. Wu, S. Hou et al., Inverse doping IrO_x_/Ti with weakened Ir-O interaction toward stable and efficient acidic oxygen evolution. Chem. **9**, 2931-2942 (2023). <https://doi.org/10.1016/j.chempr.2023.05.044>
11. K. Lee, J. Shim, H. Y. Jang, H. S. Lee, H. Shin et al., Modulating the valence electronic structure using earth-abundant aluminum for high-performance acidic oxygen evolution reaction. Chem. **9**, 3600-3612 (2023). <https://doi.org/10.1016/j.chempr.2023.08.006>
12. Z. Lu, C. Wei, X. Liu, Y. Fang, X. Hao et al., Regulating the adsorption behavior of intermediates on Ir–W@Ir–WO_3−x_ boosts acidic water oxidation electrocatalysis. Mater Chem Front. **5**, 6092-6100 (2021). <https://doi.org/10.1039/d1qm00551k>
13. Y. Xie, X. Long, X. Li, C. Chang, K. Qu et al., The template synthesis of ultrathin metallic Ir nanosheets as a robust electrocatalyst for acidic water splitting. Chem Commun. **57**, 8620-8623 (2021). <https://doi.org/10.1039/d1cc02349g>
14. M. Li, Z. Zhao, Z. Xia, M. Luo, Q. Zhang et al., Exclusive strain effect boosts overall water splitting in PdCu/Ir core/shell nanocrystals. Angew Chem Int Ed Engl. **60**, 8243-8250 (2021). <https://doi.org/10.1002/anie.202016199>
15. X. Zheng, J. Yang, P. Li, Q. Wang, J. Wu et al., Ir-Sn pair-site triggers key oxygen radical intermediate for efficient acidic water oxidation. Sci Adv. **9**, eadi8025 (2023). <https://doi.org/doi:10.1126/sciadv.adi8025>
16. J. Shan, C. Ye, S. Chen, T. Sun, Y. Jiao et al., Short-range ordered iridium single atoms integrated into cobalt oxide spinel structure for highly efficient electrocatalytic water oxidation. J Am Chem Soc. **143**, 5201-5211 (2021). <https://doi.org/10.1021/jacs.1c01525>
17. Z. Wang, P. Wang, H. Zhang, W. Tian, Y. Xu et al., Construction of hierarchical IrTe nanotubes with assembled nanosheets for overall water splitting electrocatalysis. J Mater Chem A. **9**, 18576-18581 (2021). <https://doi.org/10.1039/d1ta01839f>
18. Y. Chen, D. Liu, Q. Zhao, X. Long, J. Wang et al., IrO_x_-MoO_3_ nano-heterostructure electrocatalysts for efficient acidic water oxidation. Chem Eng J. **475**, 146255 (2023). <https://doi.org/10.1016/j.cej.2023.146255>
19. F. Zhao, B. Wen, W. Niu, Z. Chen, C. Yan et al., Increasing iridium oxide activity for the oxygen evolution reaction with hafnium modification. J. Am. Chem. Soc. **143**, 15616-15623 (2021). <https://doi.org/10.1021/jacs.1c03473>
20. W. Huo, X. Zhou, Y. Jin, C. Xie, S. Yang et al., Rhenium suppresses iridium (IV) oxide crystallization and enables efficient, stable electrochemical water oxidation. Small **19**, 2207847 (2023). <https://doi.org/10.1002/smll.202207847>
21. L.-W. Chen, F. He, R.-Y. Shao, Q.-Q. Yan, P. Yin et al., Intermetallic IrGa-IrO_x_ core-shell electrocatalysts for oxygen evolution. Nano Res. **15**, 1853-1860 (2021). <https://doi.org/10.1007/s12274-021-3778-0>
22. Y. Wang, Z. Li, L. Hou, Y. Wang, L. Zhang et al., In situ activation endows orthorhombic fluorite-type samarium iridium oxide with enhanced acidic water oxidation. ACS Appl. Mater. Interfaces **15**, 14282-14290 (2023). <https://doi.org/10.1021/acsami.2c22102>
23. Y. Pi, Y. Xu, L. Li, T. Sun, B. Huang et al., Selective surface reconstruction of a defective iridium‐based catalyst for high‐efficiency water splitting. Adv. Funct. Mater. **30**, 2004375 (2020). <https://doi.org/10.1002/adfm.202004375>
24. H. Guo, Z. Fang, H. Li, D. Fernandez, G. Henkelman et al., Rational design of rhodium-iridium alloy nanoparticles as highly active catalysts for acidic oxygen evolution. ACS Nano **13**, 13225-13234 (2019). <https://doi.org/10.1021/acsnano.9b06244>
25. H. Jin, Y. Hong, J. Yoon, A. Oh, N.K. Chaudhari et al., Lanthanide metal-assisted synthesis of rhombic dodecahedral MNi (M = Ir and Pt) nanoframes toward efficient oxygen evolution catalysis. Nano Energy **42**, 17-25 (2017). <https://doi.org/10.1016/j.nanoen.2017.10.033>
26. J. He, X. Zhou, P. Xu, J. Sun, Regulating electron redistribution of intermetallic iridium oxide by incorporating Ru for efficient acidic water oxidation. Adv Energy Mater. **11**, 2102883 (2021). <https://doi.org/10.1002/aenm.202102883>
27. J. Feng, F. Lv, W. Zhang, P. Li, K. Wang et al., Iridium-based multimetallic porous hollow nanocrystals for efficient overall-water-splitting catalysis. Adv. Mater. **29**, 1703798 (2017). <https://doi.org/10.1002/adma.201703798>
28. S. Choi, J. Park, M. K. Kabiraz, Y. Hong, T. Kwon et al., Pt dopant: Controlling the Ir oxidation states toward efficient and durable oxygen evolution reaction in acidic media. Adv. Funct. Mater. **30**, 2003935 (2020). <https://doi.org/10.1002/adfm.202003935>
29. K.-S. Kim, S.-A. Park, H.D. Jung, S.-M. Jung, H. Woo et al., Promoting oxygen evolution reaction induced by synergetic geometric and electronic effects of IrCo thin-film electrocatalysts. ACS Catal. **12**, 6334-6344 (2022). <https://doi.org/10.1021/acscatal.2c00856>
30. Z. Cheng, B. Huang, Y. Pi, L. Li, Q. Shao et al., Partially hydroxylated ultrathin iridium nanosheets as efficient electrocatalysts for water splitting. Natl. Sci. Rev. **7**, 1340-1348 (2020). <https://doi.org/10.1093/nsr/nwaa058>
31. U. Kakati, E.J. Elzinga, Z.R. Mansley, B. Roe, F. Alimohammadi et al., Iridium incorporation into MnO_2_ for an enhanced electrocatalytic oxygen evolution reaction. ChemCatChem **15**, e202201549 (2023). <https://doi.org/10.1002/cctc.202201549>
32. T. Zheng, C. Shang, Z. He, X. Wang, C. Cao et al., Intercalated iridium diselenide electrocatalysts for efficient ph-universal water splitting. Angew. Chem. Int. Ed. Engl. **58**, 14764-14769 (2019). <https://doi.org/10.1002/anie.201909369>
33. H.N. Nong, T. Reier, H.-S. Oh, M. Gliech, P. Paciok et al., A unique oxygen ligand environment facilitates water oxidation in hole-doped IrNiO_x_ core–shell electrocatalysts. Nat. Catal. **1**, 841-851 (2018). <https://doi.org/10.1038/s41929-018-0153-y>
